# Short-read RNA-seq yields lower estimates of A-to-I RNA editing levels than long-read cDNA sequencing

**DOI:** 10.1007/s44307-026-00123-w

**Published:** 2026-07-22

**Authors:** Shi Cheng, Yunling Qi, Jiaqi Ya, Lei Xia, Wenshan Zhang, Qing Xiong, Qiuli Liu, Jiaxing Zhang, Yulong Song

**Affiliations:** 1 The Affiliated Panyu Central Hospital of Guangzhou Medical University, State Key Laboratory of Respiratory Disease, GMU-GIBH Joint School of Life Sciences, Guangdong Provincial Key Laboratory of Protein Modification and Disease, The Guangdong-Hong Kong-Macau Joint Laboratory for Cell Fate Regulation and Diseases, Guangzhou Medical University, Guangzhou, Guangdong 511436 People’s Republic of China; 2 Biotherapy Center, The Third Affiliated Hospital, Sun Yat-Sen University, Guangzhou, China

**Keywords:** A-to-I RNA editing, Short-read RNA-seq, Long-read cDNA sequencing, Editing level, Quantification

## Abstract

A-to-I RNA editing, one of the most frequent RNA modifications, is essential for most aspects of RNA metabolism. However, the accuracy of quantifying A-to-I editing levels has been largely neglected, particularly when contrasting next-generation sequencing (NGS) with long-read sequencing (LRS) RNA-seq. To address it, we performed paired NGS and LRS cDNA RNA-seq of both HEK293T and U2OS cells, revealing that the A-to-I editing levels were estimated to be lower by NGS compared with LRS, a conclusion that was confirmed through full-length amplicon sequencing. Furthermore, the lower estimation of A-to-I editing levels is widespread, while we also identified this consequence using released public both same-study and cross-study NGS and LRS RNA-seq data in various human cancer cell lines. In addition, our analyses indicate that the primary factor contributing to this lower estimation is the sequencing read length difference, and a significantly positive correlation between the quantification and read length is identified by analyzing the public NGS RNA-seq data of various cell lines. We additionally found that shorter sequencing reads are more prone to aligning incorrectly to the reference genome, such as uniquely-to-multiply mapped and uniquely mapped-to-unmapped, inducing the lower estimation of A-to-I editing levels. In summary, our analyses indicate that LRS RNA-seq is preferable for accurately quantifying A-to-I editing levels.

**Supplementary Information:**

The online version contains supplementary material available at 10.1007/s44307-026-00123-w.

## Introduction

In higher eukaryotes, the diversity of transcriptomes is significantly enhanced through various RNA processes, such as alternative splicing (Bazak et al. 2014; Blencowe 2006; Johnson et al. 2003; Kampa et al. 2004; Pan et al. 2008), alternative polyadenylation (Di Giammartino et al. 2011) and RNA modifications (Boccaletto et al. 2022; Barbieri and Kouzarides 2020; Delaunay and Frye 2019; Frye et al. 2018; Zhao et al. 2020; Roundtree et al. 2017). To date, over 100 types of RNA modifications have been identified, such as *N*^6^-methyladenosine (m^6^A) and RNA editing. RNA editing is a mechanism that modifies nucleotide sequences of RNA transcripts, including base insertion, deletion and substitution (Maas and Rich 2000; Bass 2002; Farajollahi and Maas 2010; Nishikura 2010), without altering the corresponding genomic DNA (Gott and Emeson 2000). In mammals, Adenosine-to-Inosine (A-to-I) and Cytosine-to-Uracil (C-to-U) are the two most well-characterized forms of RNA editing. A-to-I editing is particularly prevalent in mammals and facilitated by the adenosine deaminases ADAR1 and ADAR2 proteins, which both act on the double-stranded (ds) RNAs (Bass 2002; Nishikura 2010; Li and Church 2013; Savva et al. 2012). The A-to-I editing is the hydrolytic deamination reaction of adenosine mediated by ADARs, and the conversion of A-to-I in RNA is essentially irreversible. In humans, there are millions of A-to-I editing sites spreading throughout the transcriptome (Bazak et al. 2014), but only a small number are found in the protein-coding regions, predominantly influenced by ADAR2, while the majority appear in noncoding regions, catalyzed by ADAR1 (Tan et al. 2017) and serve to inhibit abnormal immune responses induced by dsRNAs (Roth et al. 2019; Reautschnig et al. 2022; George et al. 2016; Liddicoat et al. 2015), especially in the repetitive regions. *Alu* repeats, which are approximately 300-nucletide-long sequences and belong to the SINE family of retrotransposons, are highly abundant in primates, with up to 1.4 million copies in the human genome, constituting nearly 10% of the human genomic DNA sequences (Lander et al. 2001). Commonly, two adjacent *Alu* repeats in reverse orientation can generate a well paired dsRNA, leading to the identification of over 99% of A-to-I editing sites located within *Alu* repeats in humans (Morse and Bass 1999; Morse et al. 2002; Levanon et al. 2004; Blow et al. 2004; Athanasiadis et al. 2004).

Inosine is recognized as guanosine (G) by cellular machinery due to their analogous chemical properties (Basilio et al. 1962; Licht et al. 2019a, b). Hence, A-to-I RNA editing significantly influences its target RNAs by inducing single amino acid substitutions in coding regions, modifying splicing sites or leading to new start or stop codons. In addition, A-to-I RNA editing also regulates RNA stability by modifying the UTRs, base-pairing abilities, folding, nuclear retention, miRNA biogenesis, miRNA targeting, RNA interference, R-loop formation, translation efficiency and the proteins associated with their targeted RNAs (Nishikura 2016, 2006; Daniel et al. 2015; Kawahara et al. 2008; Wulff and Nishikura 2012; Bass 2002, 2006; Tajaddod et al. 2016; Licht et al. 2019a, b; Jepson and Reenan 2008), and so on. Most A-to-I editing sites within the coding regions were identified in mRNAs expressed in the central nervous system. A notable example is an A-to-I editing site in the GluR-B subunit of the AMPA receptor (Sommer et al. 1991; Higuchi et al. 2000), recoding glutamine to arginine and thereby affecting the splicing efficiency of the transcript, which is necessary for the calcium permeability of the channel, with its absence resulting in lethality (Seeburg and Hartner 2003; Gaisler-Salomon et al. 2014; Veno et al. 2012). Meanwhile, the vital case of A-to-I editing in the noncoding regions is that editing can help cell distinguish “non-self” from “self” dsRNAs (Liddicoat et al. 2015). The level of A-to-I RNA editing refers to the ratio of adenosine converted to inosine. This dynamic process shows that A-to-I editing levels range from 0% to 100%, affected by environmental stimuli (Rieder et al. 2015; Duan et al. 2017) and differing among cell types (Harjanto et al. 2016; Hwang et al. 2016; Tan et al. 2017) and species (Tan et al. 2017; Sapiro et al. 2015; Zhang et al. 2017; Paz-Yaacov et al. 2010). Aberrant A-to-I editing has been linked to various human diseases, including amyotrophic lateral sclerosis (Kwak and Kawahara 2005), autism spectrum disorder (Tran et al. 2019), schizophrenia (Breen et al. 2019), epilepsy (Maas et al. 2006), depression (Tan et al. 2009; Maas et al. 2006), cancer (Han et al. 2015; Maas et al. 2001; Peng et al. 2018), dyschromatosis symmetrica hereditarian (Maas et al. 2006), and so on. Therefore, the precise measurement of RNA editing levels is essential for studying A-to-I RNA editing.

High-throughput cDNA sequencing data is commonly used to identify and quantify A-to-I RNA editing. In this process, A pairs with C, while I is transformed into G within the cDNA sequence, leading to I being represented as G during sequencing. Therefore, when the transcriptomic sequences are aligned to the reference genome, the A-to-I editing sites appear as A-to-G mismatches. And the percentage of A-to-G mismatch can serve as a metric for quantifying A-to-I RNA editing levels. Unfortunately, numerous studies have focused on developing new sequencing or bioinformatic methods identifying A-to-I editing sites (Li et al. 2011; Bahn et al. 2012; Peng et al. 2012; Kleinman and Majewski 2012; Lin et al. 2012; Pickrell et al. 2012; Piskol et al. 2013; Sakurai and Suzuki 2011; Ramaswami et al. 2013; Zhang and Xiao 2015; Ramaswami et al. 2012), but there has been limited research assessing the accuracy of quantifying A-to-I editing levels.

Over the last two decades, due to the rapid development of NGS, most studies have quantified A-to-I editing levels using NGS RNA-seq data (Picardi et al. 2010; Ju et al. 2011; Li et al. 2011; Bahn et al. 2012; Park et al. 2012). Given that the read length typically does not exceed 300 nt, the NGS is also called as short-read sequencing. Recently, the LRS technologies, also called as the third-generation sequencing, have been developed. Oxford Nanopore Technologies (ONT) and Pacific Biosciences (PacBio) are the two main representatives of the LRS platforms. Due to its lower cost, ONT is more frequently employed. Significantly different from the NGS RNA-seq, of which the RNAs are broken into small fragments firstly, the LRS RNA-seq interrogates full-length transcripts. This raises the question of whether there is a difference in the quantification of A-to-I editing levels between NGS and LRS RNA-seq. To address it, we conducted both NGS and LRS cDNA RNA-seq on HEK293T and U2OS cells in this study, revealing that the A-to-I editing levels are estimated to be lower by NGS compared with LRS.

## Materials and methods

### Cell culture

HEK293T and U2OS cells were cultured in DMEM, supplemented with 10% fetal bovine serum (Prime, Vazyme Biotech Co., Ltd) and 100 U/ml penicillin G and 100 μg/ml streptomycin at 37 °C under 5% CO_2_.

### Library construction of NGS polyA-selected and rRNA-depleted RNA-seq

HEK293T or U2OS cells were collected. Total RNAs were then extracted using the FastPure® Cell/Tissue Total RNA Isolation Kit V2 (Vazyme Biotech Co., Ltd., RC112-01). For the polyA-based mRNA enrichment, mRNAs were captured using.

VAHTS mRNA Capture Beads (Vazyme Biotech Co., Ltd., N401). For the rRNA-depleted method, ribosomal-depleted RNA was purified with Ribo-off rRNA Depletion kit (Vazyme Biotech Co., Ltd., N406). Then, the RNA-seq libraries were constructed with VAHTS Universal V8 RNA-Seq Library Prep Kit (Vazyme Biotech Co., Ltd., NRM605). Briefly, double-stranded cDNA synthesis (with dUTP), adapter ligation, and polymerase chain reaction (PCR) were performed in accordance with manufacturer protocols. Libraries with different indexs were multiplexed and loaded on an Illumina Novaseq 6000 instrument for sequencing. Three biological replicates were performed.

### LRS polyA-selected cDNA RNA-seq library construction

First, HEK293T or U2OS cells were collected. Total RNAs were then extracted using the FastPure**®** Cell/Tissue Total RNA Isolation Kit V2 (Vazyme Biotech Co., Ltd., RC112-01). The total RNAs were used as templates to synthesize the cDNA libraries using the cDNA-PCR Sequencing Kit V14 (Cat# SQK-PCS114, Oxford Nanopore Technologies, Oxford, UK). The cDNA libraries were loaded to the PromethION Flow cell (FLO-PRO114M), which was performed on the Nanopore PromethION sequencer (Oxford Nanopore Technology, Oxford, UK) at GrandOmics (Wuhan, China) for sequencing.

### Full-length DNA/RNA amplicon sequencing

Firstly, 2 μl of PCR product was detected by agarose gel electrophoresis. After qualified quality control, 200 fmol samples were taken from each PCR product. Nanopore sequencing DNA ligation library construction Kit (CW3601, CoWin) and Native Barcoding kit 96 V14 (SQK-NBD114.96, ONT) were used for library construction. Then, the library was carried out using the Qubit® 2.0 fluorescence spectrometer (Life Technologies, CA, USA) and the dsDNA HS quantitative kit (Thermo Fisher, Q33231). Sequencing was performed on the PromethION 2 Solo, with R10.4.1 flowcells (ONT FLO-PRO114M) at CWBlO Biotechnology Co. Ltd. (Taizhou, China). Fastq data was obtained using the sup4.3.0 basecall model.

### Quantification of A-to-I editing levels using NGS RNA-seq

The RNA-seq raw data was initially processed by Cutadapt (Kechin et al. 2017) (version v4.0; -q 30,30 –trim-n –length-tag 'length = ' -m 36 -e 0.1), including removing the adapter sequences from the 3' end of reads and trimming the bases with quality scores lower than 30 from both 5’ and 3’ ends. Subsequently, the clean reads were aligned to the human reference genome (GRCh37.p13) using STAR (Dobin et al. 2013) (version v2.7.10b) with the default parameters. And the corresponding Gencode v19 annotation files were obtained from UCSC table browser. Then, a custom perl script was used to calculate the editing level of each site, which is the percentage of reads with G bases in all reads. Only sites with a minimum coverage depth of 30 were retained for further analysis, and the cutoff of base quality is ≥ 30. And a human A-to-I RNA editing list compiled from our previous work (Song et al. 2020) was used, including 2,858,028 sites.

### Calculation of AEI for NGS RNA-seq data

Additionally, the AEI was calculated using the RNAEditingIndexer (Roth et al. 2019) tool with the default parameters, and the before-mentioned alignment bam files were used as the input.

### Quantification of A-to-I editing levels using LRS RNA-seq

The RNA-seq reads were aligned to the reference genome via Minimap2 (Li 2018) (version 2.17-r941; –secondary = no –cs -ax splice -uf -k14). The editing levels for the before-mentioned human A-to-I editing sites were calculated using a custom python script, with a minimum coverage depth of 30 and the minimum base quality of 7.

### Quantification of A-to-I editing levels using DNA/RNA amplicon sequencing

The fastq data was firstly aligned to the reference genome using Minimap2 with the default parameter. The editing levels for A-to-I editing sites lower estimated by NGS compared with LRS ((LRS-NGS) ≥ 10%), were calculated using a custom perl script, with a minimum coverage depth of 30 and the minimum base quality of 30.

### Removing PCR duplicate reads

The bam file was ranked by the read ID using samtools (Danecek et al. 2021) (version 1.10) sort -n, then samtools fixmate -m was employed to add mate read label. Then, rank the bam file by genome coordinate using samtools sort again. At last, mark and remove the PCR duplicates using samtools markdup -r.

### A-to-I RNA editing level quantification of unedited A candidates

The A-to-I RNA editing sites were merged, including the sites used in this study and downloaded from REDIportal database (D'Addabbo et al. 2025). Then, the least A bases in the genome were defined unedited A candidates. While numerous unedited A candidates exist, we randomly extracted 3 million sites for the further A-to-I RNA editing level quantification using the same pipeline.

### Construction of artificial NGS RNA-seq originating from the RNA-seq fastq data with 150-nt read length

As shown in Figure S13A, a custom python script was utilized to systematically construct the artificial NGS RNA-seq data including both the sequence and quality score lines from the original sequencing reads. For each artificial NGS RNA-seq with specific read length, the begging was firstly randomly chosen among the original reads, then both the following sequence and quality score with the specific length were stored into the artificial RNA-seq file, resulting in multiple RNA-seq data with different lengths. As shown in Figure S13A, during truncating reads from NGS fastq data, the beginning position was randomly chosen in the R1/R2 reads for each paired-end read, then both the corresponding length sequence and its base quality were extracted (Figure S13A). All truncated fastq files maintained the same coverage depth and strand information as the original NGS data. Each resulting fastq file with a specific read length, was processed independently in downstream analyses.

### Construction of artificial NGS RNA-seq originating from the RNA-seq bam data with 150-nt read length

As shown in Figure S13B, the alignment bam files generated from the original NGS RNA-seq fastq with 150-nt read length were used as input. Similarly, the python script was also employed to deal this issue, and the begging was firstly randomly chosen among the original reads. To ensure correct correspondence with the reference genome, CIGAR strings were rigorously recalculated based on the truncated positions. The coordinates for read starts were adjusted in accordance with the truncation start position and CIGAR operations. As shown in Figure S13B, during truncation reads from NGS bam data, the beginning position was randomly chosen in the R1/R2 reads for each paired-end read, then both the corresponding length mapping results were extracted (Figure S13B).

### Construction of artificial LRS RNA-seq originating from the RNA-seq fastq data

As shown in Figure S13C, we applied the following workflow to generate RNA-seq data of varying read lengths: (1) Randomly choosing 5’ or 3’ end as the beginning, each of the original LRS RNA-seq reads was segmented into multiple 1000-nt reads with a 50-nt sliding window. The reads shorter than 1000 nt were discarded. (2) The resultant 1000-nt fastq files were further processed using the same processed strategy as applied to NGS RNA-seq data. (3) For each resulting read-length point, the associated fastq files were analyzed separately through the LRS editing quantification pipeline to assess editing levels at recognized RNA editing sites. All fastq files with shorter read lengths had the same coverage depth as the fastq file with a read length of 1000 nt (Figure S13C).

### Construction of artificial LRS RNA-seq originating from the RNA-seq bam data

As shown in Figure S13D, the before-mentioned 1000-nt fastq files obtained from the LRS RNA-seq data, were firstly aligned to the reference genome using Minimap2 to generate a bam file. Then, similar with NGS bam data, the artificial LRS RNA-seq originating from the RNA-seq bam data was constructed, followed by the quantification of A-to-I editing levels.

### Collection and processing of public NGS and LRS RNA-seq data

As shown in Table S3, numerous NGS and LRS RNA-seq data that are publicly accessible were retrieved from the NCBI database and Oxford Nanopore Human Reference Datasets (https://github.com/nanopore-wgs-consortium/NA12878). For the analysis of public data samples with different sequencing read lengths in Fig. 3 and its corresponding supplementary figures, we applied strict quality filters and only retained samples with sequencing depth ≥ 10 million and uniquely mapping rate ≥ 60%, for subsequent editing level quantification.

### Statistical analysis

All statistical tests were performed and visualized using R (version v4.2.0) and Python (version v3.8.6). The Chi-square test was applied to calculate the p value, and the FDR (False discovery rate) was further used to assess whether an A-to-I site was significantly differently quantified between LRS and NGS RNA-seq, with a significance threshold defined as FDR < 0.05. Comparisons of global editing level distributions were performed using the Mann–Whitney U test, including comparisons between LRS and NGS RNA-seq, across different cell lines, and between polyA-selected and rRNA-depleted. The Kruskal–Wallis H test was used for multiple group comparisons. The Pearson linear correlation coefficient was applied to evaluate the relationship between two variables, such as between read length and editing level.

## Results

### The measurement of A-to-I editing levels through cDNA RNA-seq performed by NGS is significantly lower than that obtained via LRS

In consideration of the essentially biological functions and its potential application future in gene therapy, the accurate quantification of A-to-I editing is crucial. In general, cDNA RNA-seq is widely used to identify A-to-I editing sites and to quantify their levels. Due to its cost-effectiveness, almost all RNA-seq studies have relied on NGS, such as the Illumina platform. Recently, LRS have emerged, such as ONT platforms. Unlike NGS, which initially fragments RNA molecules, LRS sequences the entire RNA in its full length. This raises the question of whether the quantification of A-to-I editing through NGS RNA-seq is dependable since all RNAs are initially fragmented.

**Fig. 1:**
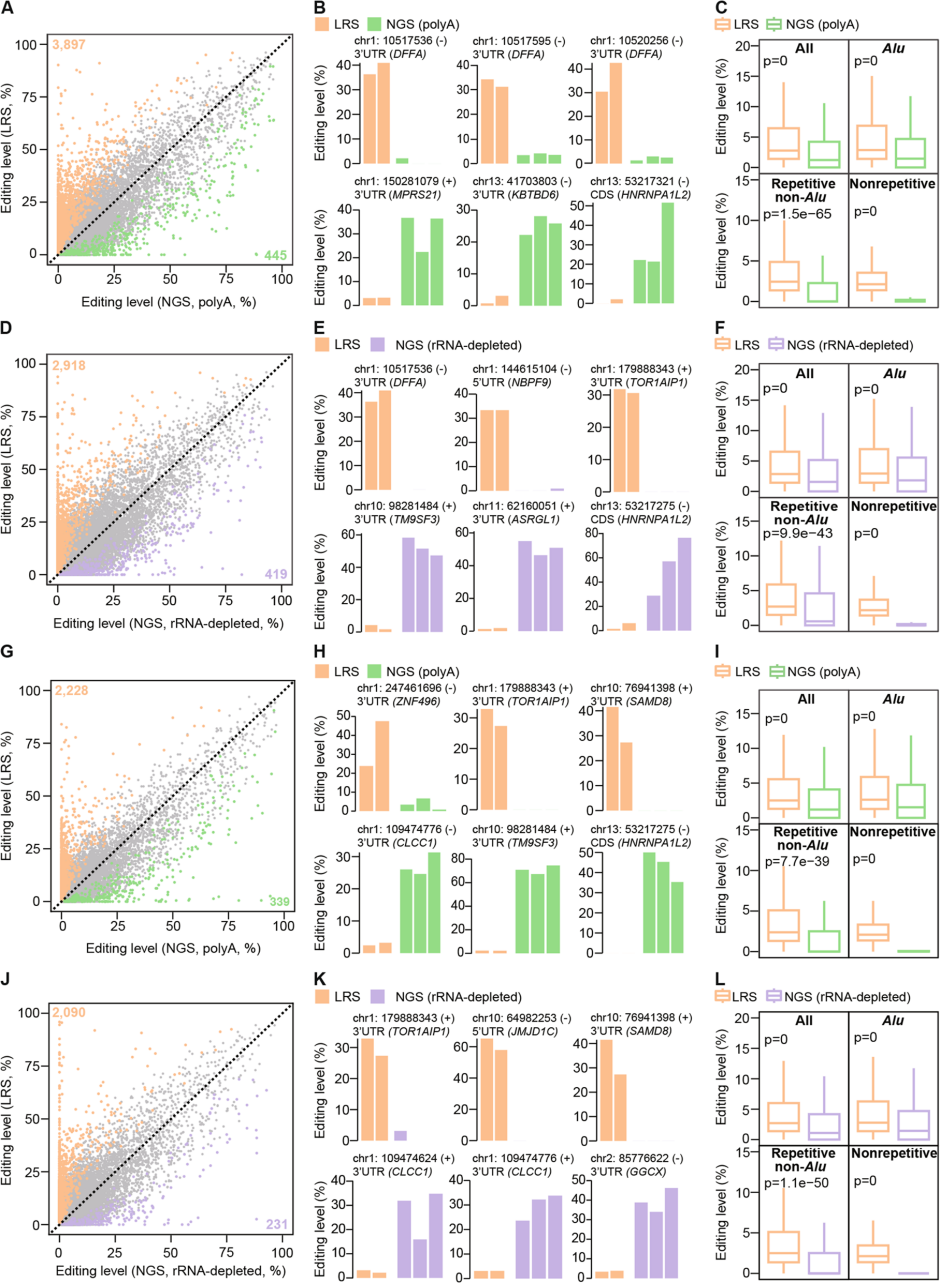
Comparison of A-to-I editing level quantification between LRS and NGS cDNA RNA-seq. **A** Dotplot of the quantification comparison of A-to-I editing levels between via LRS and NGS polyA-selected cDNA RNA-seq in HEK293T cells. **B** Cases of quantification results of several A-to-I editing sites of HEK293T cells using LRS and NGS polyA-selected cDNA RNA-seq. **C** Boxplot of the quantification comparison of A-to-I editing levels between via LRS and NGS polyA-selected cDNA RNA-seq in HEK293T cells. **D** Dotplot of the quantification comparison of A-to-I editing levels between via LRS polyA-selected and NGS rRNA-depleted cDNA RNA-seq in HEK293T cells. **E** Cases of quantification results of several A-to-I editing sites of HEK293T cells using LRS polyA-selected and NGS rRNA-depleted cDNA RNA-seq. **F** Boxplot of the quantification comparison of A-to-I editing levels between using LRS polyA-selected and NGS rRNA-depleted cDNA RNA-seq in HEK293T cells. **G** Dotplot of the quantification comparison of A-to-I editing levels between via LRS and NGS polyA-selected cDNA RNA-seq in U2OS cells. **H** Cases of quantification results of several A-to-I editing sites of U2OS cells using LRS and NGS polyA-selected cDNA RNA-seq. **I** Boxplot of the quantification comparison of A-to-I editing levels between using LRS and NGS polyA-selected cDNA RNA-seq in U2OS cells. **J** Dotplot of the quantification comparison of A-to-I editing levels between via LRS polyA-selected and NGS rRNA-depleted cDNA RNA-seq in U2OS cells. **K** Cases of quantification results of several A-to-I editing sites of U2OS cells using LRS polyA-selected and NGS rRNA-depleted cDNA RNA-seq. **L** Boxplot of the quantification comparison of A-to-I editing levels between using LRS polyA-selected and NGS rRNA-depleted cDNA RNA-seq in U2OS cells. Only the A-to-I editing site with FDR < 0.05 was defined as significant, and p values were calculated using the Mann–Whitney U test

To address it, we firstly quantified A-to-I editing levels using both NGS and LRS cDNA RNA-seq, separately, and compared their difference. Using the RNA of HEK293T cells, we conducted stranded NGS RNA-seq with the PE150 strategy on the Illumina NovaSeq 6000 platform and LRS RNA-seq on the ONT platform, both employing cDNA libraries and polyA selection (Figure S1A-B and Table S1). Surprisingly, LRS quantified 3,897 A-to-I sites significantly more highly than NGS polyA-selected RNA-seq, which identified only 445 sites with higher measurement (Fig. 1A-B), indicating LRS quantified approximately 8.8 times more sites than polyA-selected NGS RNA-seq. Furthermore, the overall A-to-I editing measurements obtained from LRS were substantially higher than those from NGS polyA-selected RNA-seq (Fig. 1C and Figure S1C-D), regardless of whether the sites were in all-inclusive, *Alu*, repetitive non-*Alu* or nonrepetitive regions. Especially, for all A-to-I editing sites, the median quantification by LRS was about 2.3 times greater than that from NGS polyA-selected RNA-seq. Even for nonrepetitive A-to-I RNA editing sites, the median quantification result via NGS polyA-selected RNA-seq is 0, which indicates no RNA editing happens, but the true median editing level is 2.1% according to LRS RNA-seq (Fig. 1C and Figure S1D).

In addition, we further conducted NGS cDNA RNA-seq using the rRNA-depleted strategy (Figure S1E) and compared the measurement of A-to-I editing level between it and LRS polyA-selected cDNA RNA-seq using the RNAs of HEK293T cells. Consistently, LRS polyA-selected cDNA RNA-seq identified 2,918 A-to-I sites significantly higher than NGS rRNA-depleted RNA-seq, which had 419 higher sites, nearly 7.0 times the count from NGS (Fig. 1D-E). Furthermore, the global quantification results of A-to-I editing by LRS RNA-seq were also significantly elevated compared to NGS rRNA-depleted cDNA RNA-seq (Fig. 1F and Figure S1F-G), regardless of whatever the sites were in *Alu*, repetitive non-*Alu* or nonrepetitive region. Consistently, for all sites, the median quantification via LRS was nearly 1.8 times that of NGS rRNA-depleted RNA-seq. Meanwhile, for nonrepetitive sites, the median measurement result from NGS rRNA-depleted RNA-seq is also 0%, representing nearly no RNA editing happening, but the actual median editing level should be 2.2% according to LRS (Fig. 1F and Figure S1G). Collectively, these results indicate that NGS cDNA RNA-seq tends to lower estimate A-to-I editing levels when compared to LRS.

To further validate the findings, we conducted additional NGS and LRS RNA-seq using U2OS cells and re-evaluated the results (Figure S2). Expectedly, LRS RNA-seq identified 2,228 A-to-I sites with significantly higher quantification, nearly 6.6 times more than the NGS polyA-selected RNA-seq (Fig. 1G-H). Additionally, the overall results from LRS were also found significantly higher than those obtained from NGS polyA-selected RNA-seq (Fig. 1I and Figure S2C-D). Furthermore, LRS revealed 2,090 A-to-I sites that were significantly more quantified, approximately 9.0 times greater than those found in NGS rRNA-depleted RNA-seq (Fig. 1J-K), with the overall measurement results from LRS also being significantly higher than NGS rRNA-depleted RNA-seq (Fig. 1L and Figure S1F-G). Collectively, these results demonstrate that the A-to-I editing levels were globally lower estimated by NGS RNA-seq compared to LRS.

### The lower estimates of A-to-I editing level by NGS RNA-seq than LRS are proved by full-length DNA/RNA amplicon sequencing

**Fig. 2:**
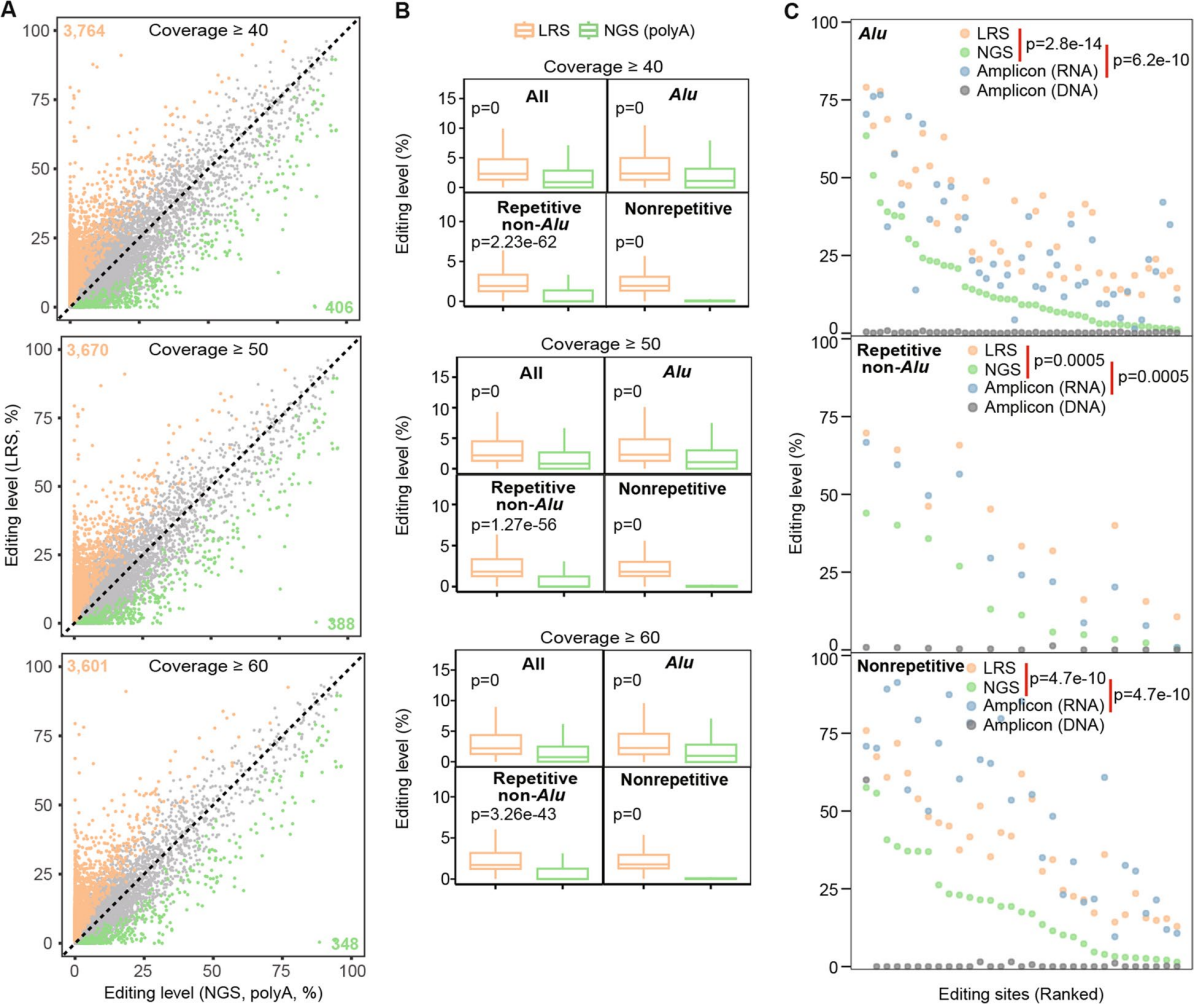
Validation of the A-to-I RNA editing level variance between LRS and NGS cDNA RNA-seq using amplicon sequencing. **A-B** Dotplot (**A**) and boxplot (**B**) of the quantification comparison of A-to-I editing levels between via LRS and NGS polyA-selected cDNA RNA-seq in HEK293T cells with different coverage filter. **C** Dotplot of the quantification comparison of A-to-I editing levels between via LRS polyA-selected, NGS rRNA-depleted cDNA RNA-seq, DNA and RNA amplicon sequencing in HEK293T cells, and p values were calculated using the one-tailed Wilcoxon Signed‑Rank test. Only the A-to-I editing site with FDR < 0.05 was defined as significant, and p values were calculated using the Mann–Whitney U test

To provide additional evidence supporting our findings that A-to-I editing levels were lower estimated by NGS RNA-seq than LRS, series of analyses and experiments were further employed. First of all, when raising the coverage from 30 (Fig. 1A) to 40, 50 and 60 (Fig. 2A), more significantly highly quantified A-to-I RNA editing sites by LRS were still observed than NGS polyA-selected RNA-seq in HEK293T, with fold changes ranging from 9.3 to 10.3. And the consistent observations were observed between the pair of LRS and NGS rRNA-depleted RNA-seq in HEK293T (Figure S3A-B), and the pairs of LRS and NGS RNA-seq in U2OS (Figure S3C-F). Secondly, in the previous comparison, the base quality of LRS was set to ≥ 7, a standard practice in LRS analyses, we further raised the cutoff as 10 and re-analyzed the data. Consistently, the findings indicated lower estimates of A-to-I RNA editing levels by NGS compared to LRS (Figure S4). Thirdly, while all the PCR duplicate reads were saved for analyses, to test whether PCR duplicates affect the LRS/NGS difference, we removed the PCR duplicates of both NGS and LRS RNA-seq (see Methods), and lower estimates of A-to-I editing levels identified through NGS were still observed compared to those obtained from LRS (Figure S5). Fourthly, as a control, no significant difference in quantification was observed between NGS polyA-selected and rRNA-depleted RNA-seq (Figure S6A-D), or between HEK293T and U2OS cell lines (Figure S7). Fifthly, the coverage of LRS used for quantifying A-to-I RNA editing levels was significantly high, with a median close to 100 (Figure S6E). Sixthly, as a control, all the quantification results of A-to-I RNA editing of unedited A candidates using LRS/NGS were nearly 0% (Figure S8), reflecting the A-to-I RNA editing level quantification results of LRS/NGS RNA-seq are reliable.

Last but not the least, to supply the direct evidence, the full-length DNA/RNA amplicon sequencing was conducted (see Methods). Some *Alu*, repetitive non-*Alu* and nonrepetitive A-to-I RNA editing sites, which were estimated to be lower by NGS compared to LRS RNA-seq, were selected for these experiments (Table S2). As anticipated, the quantification of A-to-I editing levels through full-length DNA amplicon sequencing was found to be nearly 0%, and the measurement results of both LRS and full-length RNA amplicon sequencing were significantly higher compared to NGS (Fig. 2C). Collectively, these observations demonstrated that the A-to-I RNA editing levels were lower estimated by NGS when compared to LRS.

### The disparity in the quantification of A-to-I editing level between LRS and NGS RNA-seq is significantly greater than that observed among different cell lines

Our analyses demonstrate that A-to-I editing levels were lower estimated by NGS RNA-seq than LRS, but how to describe the scale of the quantification difference? To address it, we counted the variations between LRS and NGS, NGS polyA-selected and rRNA-depleted RNA-seq, HEK293T and U2OS cell lines. First of all, the number of significantly highly quantified A-to-I editing sites is 6.6 to 9.0 times higher in LRS than that in NGS (Fig. 1A, D, G and J), indicating the largest discrepancy, while the fold change between polyA-selected and rRNA-depleted RNA-seq is merely 0.6 to 4.1 (Figure S6A and S6C). In comparison, for the LRS, NGS polyA-selected and rRNA-depleted RNA-seq, the fold differences between HEK293T and U2OS cells range from 0.4 to 2.5 (Figure S7A-C).

Furthermore, when considering all A-to-I editing sites, the median fold differences in quantification between LRS and NGS RNA-seq are at their highest, ranging from 1.8 to 2.5 (Fig. 1C, F, I and L). In contrast, the differences observed between NGS polyA-selected and rRNA-depleted RNA-seq are relatively lower, falling between 0.9 and 1.3 (Figure S6B and S6D). The comparison between HEK293T and U2OS cells yields a fold range of 0.9 to 1.1 for LRS, NGS polyA-selected and rRNA-depleted RNA-seq (Figure S7D-F). Notably, for nonrepetitive A-to-I RNA editing sites, all the median quantification results for NGS RNA-seq are 0%, indicating virtually no RNA editing, while LRS RNA-seq shows median quantification results of 2.1% to 2.2% (Fig. 1C, F, I and L). Nevertheless, there is no significantly directional difference whatever between NGS polyA-selected and rRNA-depleted RNA-seq (Figure S6B and S6D) or between two distinct cell lines (Figure S7D-F).

In addition, we investigated whether alignment parameters influence the measurement results of A-to-I editing levels. There are three different alignment strategies: a) utilizing only uniquely mapped hits; b) combining uniquely mapped hits with one of the multiply mapped hits that has the highest mapping scores; c) employing both the uniquely mapped hits and all the multiply mapped hits with the best mapping scores. To address it, NGS polyA-selected (Figure S9A), rRNA-depleted (Figure S9B) and LRS (Figure S9C) RNA-seq of HEK293T cell lines, were utilized to quantify A-to-I editing levels based on those three different alignment strategies. Expectedly, no significantly directional difference was detected between the alignment strategies and measurement results of A-to-I editing levels (Figure S9A-C). In addition, the similar results were identified across all three types of RNA-seq from U2OS cell lines (Figure S9D-F). Taken together, these results demonstrate that the differences in quantifying A-to-I editing levels between LRS and NGS RNA-seq are substantial and exceeds the variability among different cell lines.

### The results of quantifying A-to-I editing level through NGS RNA-seq show a positive correlation with read length

Our analyses demonstrate that the global quantification results of A-to-I editing achieved through LRS RNA-seq are significantly higher than that obtained via NGS. This discrepancy is likely attributable to the influence of read length on the measurement of A-to-I editing level, while RNA molecules are firstly fragmented in NGS, unlike in LRS. Expectedly, the median clean read lengths for LRS RNA-seq performed in this study ranged from 812 to 878 nt (Figure S10A-B), in contrast to the shorter lengths of approximately 150 nt observed with NGS (Figure S10C-F). To investigate the impact of read length on quantifying A-to-I editing levels further, we analyzed the correlation between the measurement results and read lengths of 50, 75, 100, or 150 nt using NGS RNA-seq.

**Fig. 3:**
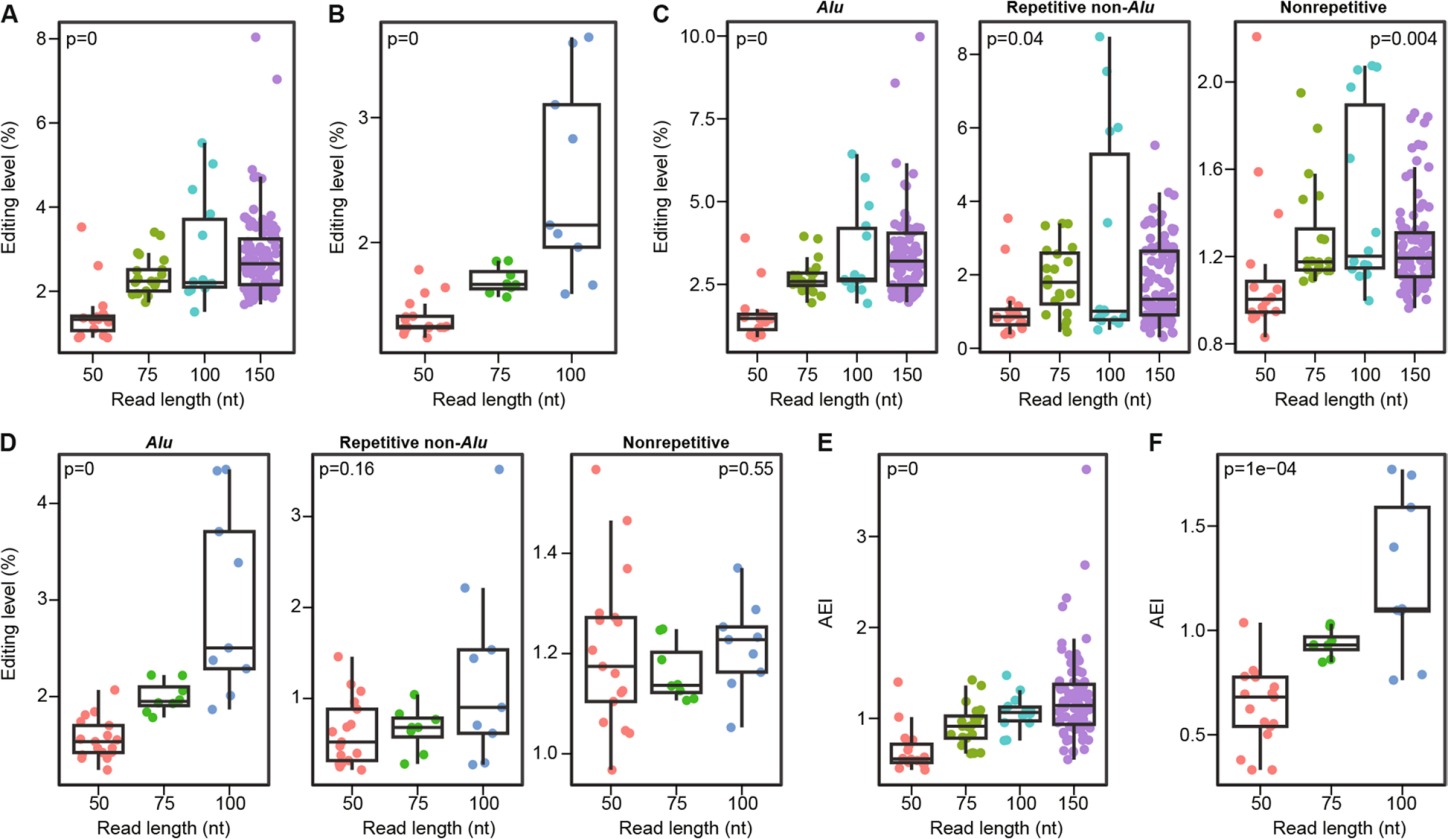
Comparison of quantification for A-to-I editing levels across various read lengths in NGS cDNA RNA-seq conducted on HEK293T cells. **A-B** The quantification comparison of A-to-I editing levels among different read lengths of paired-end (**A**) or single-end (**B**) NGS cDNA RNA-seq in HEK293T cells. **C-D** The quantification comparison of A-to-I editing levels located in *Alu*, repetitive non-*Alu* or nonrepetitive region among different read length of paired-end (**C**) or single-end (**D**) NGS cDNA RNA-seq in HEK293T cells. **E–F** The quantification comparison of AEI among different read lengths of paired-end (**E**) or single-end (**F**) NGS cDNA RNA-seq in HEK293T cells. For all analyses, each dot represents one RNA-seq sample; for each sample, the average A-to-I editing level (**A**-**D**) or AEI (**E**–**F**) was used. P values were calculated using the Kruskal–Wallis H test

Initially, we gathered all publicly available NGS RNA-seq datasets from HEK293T cells with varying read lengths (Table S3). The read lengths for NGS paired-end RNA-seq included 50, 75, 100, and 150 nt (Fig. 3A). The average A-to-I editing level for each sample was employed in this analysis. Intriguingly, a significantly positive correlation was observed between measurement results of A-to-I editing level and read lengths, with the lowest editing levels found in the samples with 50-nt read length, followed by 75-nt, 100-nt, and the highest levels in the 150-nt samples (Fig. 3A). Then, we analyzed the NGS single-end RNA-seq data from HEK293T cells. Expectedly, the quantification of A-to-I editing exhibited a positive correlation with read length (Fig. 3B).

To further explore the correlation between quantification results and read lengths in HEK293T NGS RNA-seq, the A-to-I editing sites were divided into three groups: *Alu*, repetitive non-*Alu* and nonrepetitive. First, for the *Alu* group, which encompasses nearly all A-to-I editing sites, we found a significantly positive correlation with read length in both NGS paired-end and single-end RNA-seq data from HEK293T cells (Fig. 3C-D). Additionally, a positive correlation was observed in both repetitive non-*Alu* and nonrepetitive A-to-I RNA editing sites with NGS paired-end RNA-seq (Fig. 3C-D), rather than single-end data, although the median quantification of 100-nt is higher than that of 50- or 70-nt for single-end data.

Then, while *Alu* editing index (AEI) was developed as a robust and simple-to-use quantification tool (Roth et al. 2019), which could be used to map the global A-to-I editing level of human samples, we next quantified the A-to-I editing level using the AEI and analyzed the correlation between the measurement result and read length using NGS RNA-seq in HEK293T cells again. Consistently, both NGS paired-end (Fig. 3E) and single-end (Fig. 3F) RNA-seq revealed a significant positive correlation between the AEI and read length. At last, we further explored the relationship between the measurements of A-to-I editing level and read length using NGS RNA-seq in additional custom cell lines, such as U2OS, HeLa, HepG2, K562, A549 and U87 (Figure S11 and S12). Consistently, whatever using average A-to-I editing level (Figure S11) or AEI (Figure S12), the quantification results demonstrated a significant positive correlation with read length for both NGS paired-end and single-end RNA-seq across nearly all these cell lines. Taken together, these analyses demonstrate that NGS RNA-seq quantification of A-to-I editing levels is influenced by read length, demonstrating a positive relationship between these two variables.

### The results of measuring A-to-I editing levels show a positive correlation with read length when using artificial NGS and LRS RNA-seq

By analyzing the NGS RNA-seq data from custom human cell lines sourced from a public database, we established that read length affects the quantification result of A-to-I editing level using NGS RNA-seq while the data with longer read gains higher editing level. Given that the released NGS RNA-seq was conducted by various laboratories and different sequencing platforms, which may affect the observed correlation between read length and measurement result of A-to-I editing level. Thus, we constructed the artificial NGS or LRS RNA-seq data based on the NGS or LRS RNA-seq conducted in this study and detected the correlation between read length and quantification result of A-to-I editing level quantification.

**Fig. 4:**
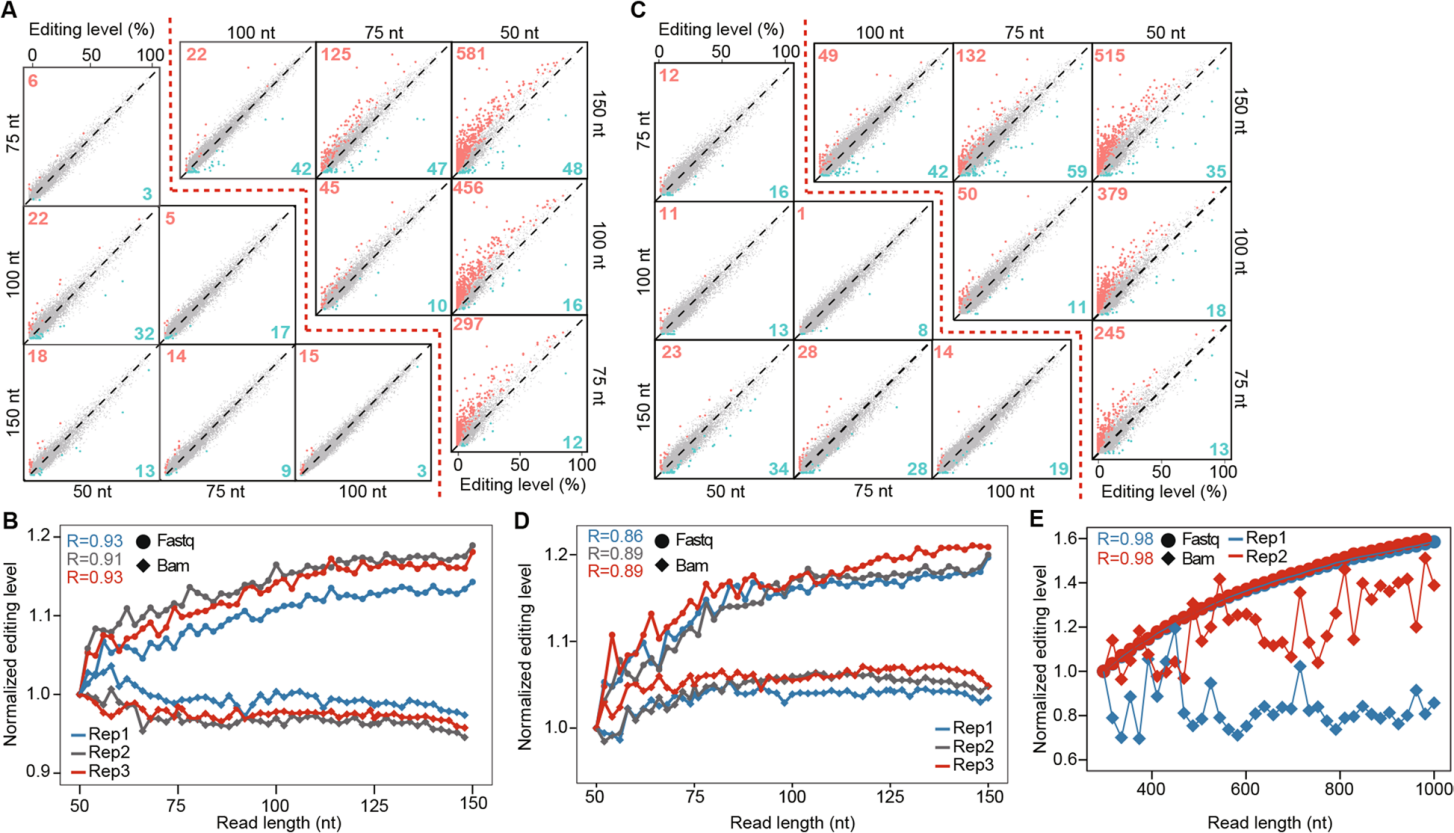
Assessment of A-to-I editing level quantification across different read lengths in artificial NGS or LRS RNA-seq within the HEK293T cell line. **A** Dotplot of the quantification comparison of A-to-I editing levels between artificial NGS polyA-selected cDNA RNA-seq with different read lengths in HEK293T cells. The upper-right represents the artificial RNA-seq derived from the fastq, while the bottom-left represents the artificial RNA-seq derived from the bam. **B** The correlation between the normalized quantification of A-to-I editing levels and the read lengths of artificial NGS polyA-selected cDNA RNA-seq in HEK293T cells. **C** Dotplot of the quantification comparison of A-to-I editing levels between artificial NGS rRNA-depeted cDNA RNA-seq with different read lengths in HEK293T cells. The upper-right represents the artificial RNA-seq derived from the fastq, while the bottom-left represents the artificial RNA-seq derived from the bam. The three replicates were merged. **D** The correlation between the normalized quantification of A-to-I editing levels and the read lengths of artificial NGS rRNA-depleted cDNA RNA-seq in HEK293T cells. The editing level of each sample was normalized by dividing editing level of the RNA-seq with 50-nt read length. **E** The correlation between the normalized quantification of A-to-I RNA editing levels and the read lengths of artificial LRS polyA-selected cDNA RNA-seq in HEK293T cells. The editing level of each sample was normalized by dividing editing level of the RNA-seq with 297-nt read length. P values were calculated using the Mann–Whitney U test and p value < 0.05 was defined as significant

Initially, we randomly extracted the paired-end RNA-seq data of 50, 75 and 100 nt from the 150-nt NGS polyA-selected RNA-seq of HEK293T cells (Figure S13A-B), which was conducted in this study (Table S1). Intriguingly, through paired comparisons, we discovered that RNA-seq with longer read lengths identified significantly more highly quantified A-to-I editing sites, except in the comparison between the 150-nt and 100-nt RNA-seq (Fig. 4A, upper-right). The increase in the number of significantly highly quantified A-to-I editing sites between longer and shorter reads ranged from 2.7 to 28.5 (Fig. 4A, upper-right), such as 12.1 times between 150-nt and 50-nt and 24.8 times between 75-nt and 50-nt, excluding the pair involving 150-nt and 100-nt RNA-seq. Furthermore, we randomly extracted 50 NGS paired-end polyA-selected RNA-seq data from HEK293T cells from the 150-nt fastq with the step of 2 nt and calculated the correlation between read length and A-to-I editing level quantification, revealing a strongly positive correlation (Fig. 4B). As expected, similar results were obtained using NGS rRNA-depleted RNA-seq from HEK293T cells (Fig. 4C, upper-right, and 4D). Consistently, we also detected a strong positive correlation between read length and quantification of A-to-I editing level in both NGS polyA-selected (Figure S14A, upper-right, and S14B) and rRNA-depleted (Figure S14C, upper-right, and S14D) RNA-seq data from U2OS cells.

At last, the LRS RNA-seq data from both HEK293T and U2OS cells was also analyzed. To improve the sequencing depth, the LRS RNA-seq was initially segmented into 1000-nt single-end RNA-seq using a 50-nt sliding window, followed by the random extraction of artificial RNA-seq with varying read lengths from the 1000-nt fastq data (Figure S13C-D). Consistently, a strong positive correlation between read length and the quantification of A-to-I editing levels was also observed in the 38 artificial LRS RNA-seq data of both HEK293T (Fig. 4E) and U2OS (Figure S14E) cells. Collectively, these findings demonstrate that read length has a significant effect on the quantification of A-to-I editing levels in RNA-seq, with longer reads resulting in higher quantification outcomes.

### Inaccurate alignment contributes to the influence of read length on the quantification of A-to-I editing levels using RNA-seq

Our analysis reveals that read length affects the quantification results for A-to-I editing levels in NGS RNA-seq, while the RNA molecules are firstly broken into pieces. The primary reason is that read length of RNA-seq may affect the alignment accuracy, with shorter reads potentially being misaligned to the reference, consequently affecting the quantification of A-to-I editing level. To test the hypothesis, we randomly constructed the artificial RNA-seq with shorter read lengths from the NGS polyA-selected RNA-seq of HEK293T using two distinct approaches (Fig. 4A and Figure S13A-B). One is originating from the fastq data, then mapping to the reference and quantifying the A-to-I editing level (Fig. 4A, upper-right, and Figure S13A), while the other is originating from the bam data and then directly quantifying (Fig. 4A, bottom-left, and Figure S13B). Expectedly, only a small number of significantly different A-to-I editing sites were identified between the paired RNA-seq datasets with different read lengths derived from the bam (Fig. 4A, bottom-left) compared to those from the fastq (Fig. 4A, upper-right). In addition, the quantification results for A-to-I editing levels were consistent across the artificial NGS polyA-selected RNA-seq with different read lengths derived from the bam in HEK293T (Fig. 4B), whereas a strong positive correlation was observed in the fastq-derived data (Fig. 4B). Consistently, similar findings were also observed in the artificial NGS rRNA-depleted RNA-seq of HEK293T (Fig. 4C-D), the artificial NGS polyA-selected and rRNA-depleted RNA-seq of U2OS (Figure S14A-D), and the artificial LRS RNA-seq for both HEK293T (Fig. 4E and Figure S13C-D) and U2OS (Figure S14E). These outcomes demonstrate that the alignment accuracy is a key factor in how read length influences the quantification of A-to-I editing levels.

**Fig. 5:**
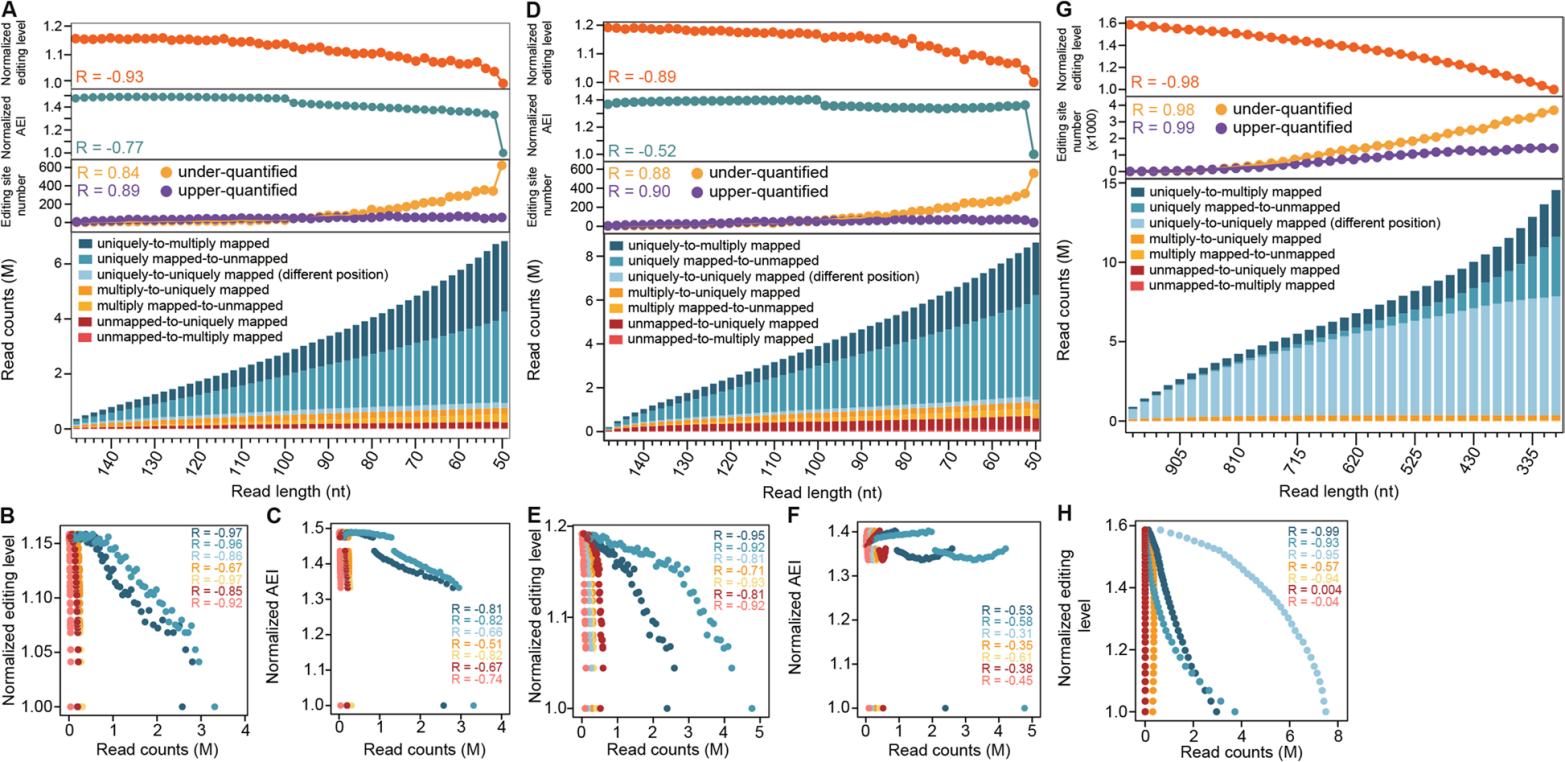
The correlation between A-to-I editing level quantification and inaccurate alignment types among different read lengths of artificial NGS or LRS RNA-seq in the HEK293T cell line.** A** The normalized editing level, AEI, significantly under- or upper-quantified editing site number and inaccurately aligned read count among the read lengths of artificial NGS polyA-selected cDNA RNA-seq of HEK293T cells. **B** The correlation between normalized editing level and inaccurately aligned read count of artificial NGS polyA-selected cDNA RNA-seq of HEK293T cells. **C** The correlation between normalized AEI and inaccurately aligned read count of artificial NGS polyA-selected cDNA RNA-seq of HEK293T cells. **D** The normalized editing level, AEI, significantly under- or upper-quantified editing site number and inaccurately aligned read count among the read lengths of artificial NGS rRNA-depleted cDNA RNA-seq of HEK293T cells. **E** The correlation between normalized editing level and inaccurately aligned read count of artificial NGS rRNA-depleted cDNA RNA-seq of HEK293T cells. **F** The correlation between normalized AEI and inaccurately aligned read count of artificial NGS rRNA-depleted cDNA RNA-seq of HEK293T cells. **G** The normalized editing level, significantly under- or upper-quantified editing site number and inaccurately aligned read count among the read lengths of artificial LRS polyA-selected cDNA RNA-seq of HEK293T cells. **H** The correlation between normalized editing level and inaccurately aligned read count of artificial LRS polyA-selected cDNA RNA-seq of HEK293T cells

To further explore how alignment affects quantifying A-to-I editing levels in NGS RNA-seq with varying read lengths, several analyses were conducted. First, we compared the quantification of A-to-I editing levels and alignment results between NGS polyA-selected RNA-seq with a read length of 150 nt from HEK293T and its corresponding artificial RNA-seq with different read lengths (Fig. 5A and Figure S13A). Intriguingly, a significantly positive correlation was observed between the A-to-I editing level measurements (Fig. 5A, upper) or the AEI (Fig. 5A, second) and read length. Compared with the NGS RNA-seq with 150-nt length, the number of A-to-I sites that were significantly under-quantified increased as the read length decreased (Fig. 5A, third). We identified two inflection points for the A-to-I editing level, the AEI, and the number of sites, one near 100 nt and another around 50 nt. In addition, we compared the differences in alignment results of RNA-seq samples between those with 150 nucleotides and shorter lengths (Fig. 5A, bottom). Intriguingly, we found that shorter reads tended to align inaccurately to the reference genome, especially those that were uniquely-to-multiply mapped and uniquely mapped-to-unmapped. As read lengths decreased, there was a significant increase in the read counts for both uniquely-to-multiply mapped and uniquely mapped-to-unmapped, with counts reaching several million (Fig. 5A, bottom). A significant negative correlation was found between the counts of uniquely-to-multiply mapped or uniquely mapped-to-unmapped reads and the A-to-I editing levels (Fig. 5B) or the AEI (Fig. 5C). These findings indicate that shorter reads of RNA-seq are more susceptible to misalignment to incorrect positions on the reference genome, leading to a lower estimate of the A-to-I editing level.

Second, we explored the NGS polyA-selected RNA-seq of U2OS, which yielded comparable results (Figure S15A-C). In addition, the consistent results were identified in the NGS rRNA-depleted RNA-seq analyses for both HEK293T (Fig. 5D-F) and U2OS (Figure S15D-F). We examined the artificial LRS RNA-seq for both HEK293T (Fig. 5G-H) and U2OS (Figure S15G-H). A comparison of the artificial LRS RNA-seq with shorter read lengths against the 1000-nt data revealed a notable reduction in A-to-I editing levels in HEK293T (Fig. 5G, upper). And the number of A-to-I sites that were significantly under-quantified increased as the read length decreased (Fig. 5G, middle). Consistently with the NGS RNA-seq, we also observed that shorter reads tended to align incorrectly to the reference genome. While there were numerous reads that were uniquely mapped to multiple positions and those uniquely mapped to unmapped regions, the majority were reads that uniquely aligned to the reference genome, but the shorter reads were mapped to the different positions of the reference compared to 1000-nt reads (Fig. 5G, bottom, and 5H). Similar trends were also identified in U2OS (Figure S15G-H). Collectively, these findings demonstrate that read length in RNA-seq affects the alignment accuracy, leading to a lower estimation of A-to-I editing levels when using short-read NGS RNA-seq.

### Lower estimates of A-to-I editing level by NGS RNA-seq is a common issue, compared with LRS

Our analysis indicates that the level of A-to-I editing detected by NGS RNA-seq is lower than that obtained from LRS, primarily due to alignment inaccuracies. Shorter reads tend to be misaligned to incorrect locations on the reference genome. Since both NGS and LRS RNA-seq were performed on HEK293T and U2OS cell lines, we aimed to evaluate the degree of this underestimation across different datasets. We subsequently analyzed additional paired NGS and LRS cDNA RNA-seq dataset with high sequencing depth and produced by the same laboratory.

**Fig. 6:**
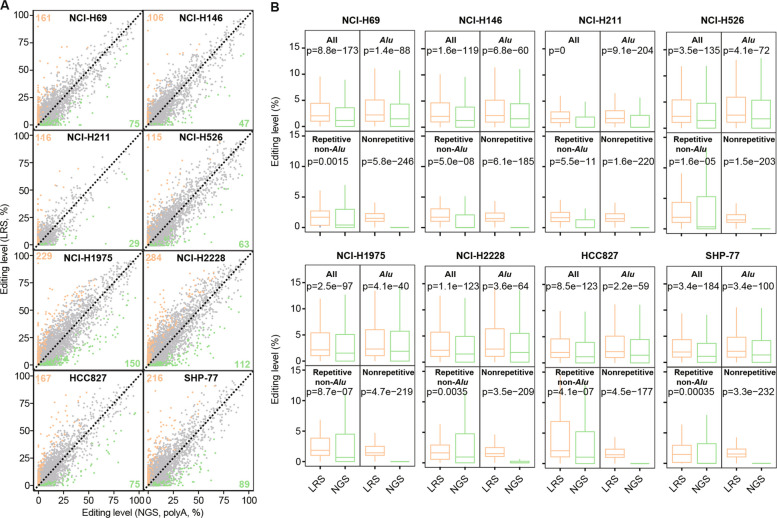
Comparison of A-to-I editing level quantification between same-study LRS and NGS cDNA RNA-seq in a range of human lung cancer cell lines. Dotplot (**A**) and boxplot (**B**) of the quantification comparison of A-to-I editing levels between LRS and NGS cDNA RNA-seq of eight lung cancer cell lines. Only the A-to-I editing site with FDR < 0.05 was defined as significant. P values were calculated using the Mann–Whitney U test

The dataset (GEO accession: GSE303762) performed the paired NGS and LRS polyA-selected cDNA RNA-seq from eight human lung cancer cell lines, including NCI-H69, NCI-H146, NCI-H211, NCI-H526, NCI-H1975, NCI-H2228, HCC827 and SHP-77 (Table S4). Consistently with our observations in both HEK293T (Fig. 1A) and U2OS (Fig. 1C), for all these eight cell lines, LRS identified more significantly highly quantified A-to-I sites in all eight cell lines, than NGS RNA-seq, with an increase factor ranging from 1.5 to 4.0 (Fig. 6A). Furthermore, LRS generated substantially higher global quantification of A-to-I editing across all categories of sites, including all the sites whatever located in *Alu*, repetitive non-*Alu* or nonrepetitive region, than NGS RNA-seq. Specifically, all the A-to-I editing levels of nonrepetitive sites were quantified as 0% by NGS RNA-seq, while LRS provided quantification results of nearly 2% (Fig. 6B).

**Fig. 7:**
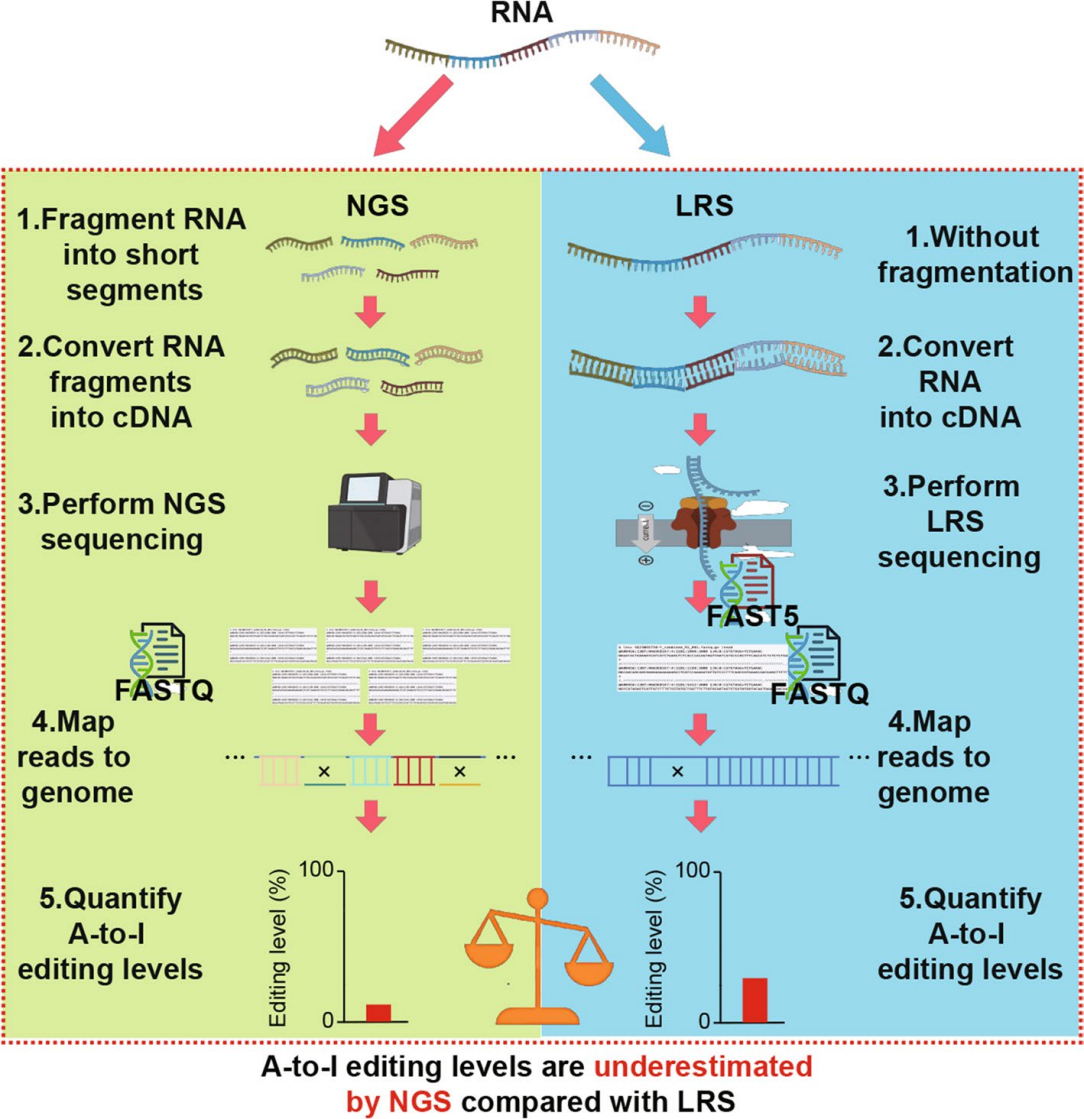
Schematic diagram of A-to-I editing level underestimated by NGS compared with LRS RNA-seq

Next, we analyzed the LRS cDNA RNA-seq data from the GM12878 cell line (https://github.com/nanopore-wgs-consortium/NA12878, Table S3). Although there was no corresponding NGS cDNA RNA-seq data within the same study, five public NGS cDNA RNA-seq dataset (GEO accession: GSE78553 (Consortium 2012), GSE78554 (Consortium 2012), GSE78555 (Consortium 2012), GSE90222 (Consortium 2012), GSE90233 (Consortium 2012), Table S5) from the ENCODE project provided adequate sequencing depth. Majority of the sites with significant changes in the quantification of A-to-I editing levels were found highly quantified via LRS than NGS RNA-seq (Figure S16A-C), whatever located in *Alu*, repetitive non-*Alu* or nonrepetitive region, while 16,727 sites are significantly higher via LRS and only 704 by NGS, with the fold as 23.8 (Figure S16A). Then, the LRS cDNA RNA-seq of HeLa was also downloaded (GEO accession: GSE277764 (Liu et al. 2025), Table S5) for additional analysis, selecting four NGS cDNA RNA-seq dataset with substantial sequencing depth at random (GEO accession: GSE112007 (Li et al. 2019), GSE173171 (Bizarro et al. 2021), GSE183535 (Gill et al. 2021), GSE186370 (Batie et al. 2022), Table S5). Consistently, we observed that A-to-I editing levels were significantly more quantified via LRS than by NGS RNA-seq (Figure S16D-F), including sites situated in *Alu*, repetitive non-*Alu*, or nonrepetitive regions, with 7,020 A-to-I editing sites showing higher quantification via LRS compared to 1,099 by NGS, resulting in a fold change of 6.4 (Figure S16D). In summary, our findings demonstrate that NGS RNA-seq frequently lower estimates A-to-I editing levels when compared to LRS (Fig. 7).

## Discussion

As a process only altering the RNA sequence without affecting its reference genome, RNA editing is a ubiquitous epigenetic mechanism appearing in nearly 85% of the human transcriptome, playing a key role in RNA metabolisms (Hartner et al. 2009; Nishikura 2016; Teoh et al. 2018). The most pervasive form of RNA editing in human is A-to-I RNA editing (Nishikura 2016), while ~16 millions of putative A-to-I editing sites in human were collected in REDIportal database (D'Addabbo et al. 2025). With the structural similarities, cellular machines recognize I base as G base (Basilio et al. 1962; Licht et al. 2019a, b), hence, A-to-I RNA editing has types of regulation on its RNA substrates, including changing coding, folding, base-pairing, splicing, translation efficiency, proteins associated with the targeted RNAs (Licht et al. 2019a, b; Licht et al. 2019a, b; Bass 2002; Tajaddod et al. 2016; Nishikura 2016), and so on. The A-to-I editing levels exhibit spatial–temporal dynamics, including increasing during development and differing across different tissues (Licht et al. 2019a, b; Wahlstedt et al. 2009; Li et al. 2018; Tan et al. 2017; Huntley et al. 2016). Aberrant A-to-I editing level has been associated with numerous human diseases, for example, tumorigenesis in various cancer types (Teoh et al. 2018; Galeano et al. 2012; Paz-Yaacov et al. 2015; Han et al. 2015; Zipeto et al. 2016) and diseases of the central nervous system (Maas et al. 2006), such as amyotrophic lateral sclerosis (Kwak and Kawahara 2005), depression, epilepsy, schizophrenia. Hence, accurately quantifying A-to-I editing level is vital in this field, which has been overlooked for a long time.

Here, we performed the NGS polyA-selected and rRNA-depleted, and LRS polyA-selected cDNA RNA-seq of both HEK293T and U2OS cell lines, consequently found that the A-to-I editing levels were globally lower estimated by NGS RNA-seq compared to LRS. The discrepancy in quantification of A-to-I editing level between LRS and NGS RNA-seq was significantly larger than the difference among different cell lines, for example, the folds of significantly more highly quantified A-to-I editing site number between LRS and NGS RNA-seq are from 6.6 to 9.0 (Fig. 1A, D, G and J), as only a range of 0.6 to 4.1 between NGS polyA-selected and rRNA-depleted cDNA RNA-seq (Figure S6A and S6C), only ranging from 0.4 to 2.5 between HEK293T and U2OS cells (Figure S7A-C). Furthermore, for all A-to-I editing sites, the median quantification ratios between LRS and NGS RNA-seq ranged from 1.8 to 2.5 (Fig. 1C, F, I and L), whereas the range for NGS polyA-selected and rRNA-depleted cDNA RNA-seq was from 0.9 to 1.3 (Figure S6B and S6D), and only ranging from 0.9 to 1.1 between HEK293T and U2OS cells (Figure S7D-F). Especially, for nonrepetitive A-to-I RNA editing sites NGS RNA-seq recorded a median quantification of 0%, indicating nearly no RNA editing, but LRS RNA-seq showed a median quantification of 2.1% to 2.2% (Fig. 1C, F, I and L). Nevertheless, no observable differences were detected between NGS polyA-selected and rRNA-depleted RNA-seq (Figure S6B and S6D) or between the two cell lines (Figure S7D-F). Our analyses reflect that the A-to-I editing level is estimated lower by NGS RNA-seq compared to LRS, and further analyses reflect that the low estimation is a common issue across various human cell lines. Our findings advocate for the use of LRS RNA-seq for a more accurate quantification of A-to-I editing levels. Considering that the cost of LRS is considerably greater than that of NGS, researchers might still prefer NGS for their investigations due to the distinct A-to-I editing sites, especially the sites located at repetitive regions, additional sanger sequencing and experiments are advisable to verify their validity or only using the uniquely mapped reads or raising the coverage depth to quantify their levels. In addition, dUTP stranded library strategy was employed for NGS RNA-seq in this study, which enhances alignment accuracy and allows for differentiation between reads from overlapping sense and antisense genes. While hg19 human genome was employed in this study, to check whether the genome version affects the LRS/NGS difference on A-to-I RNA editing level quantification, we reanalyzed LRS and NGS polyA-selected RNA-seq of HEK293T using GRCh38 and found the consistent results (Figure S17).

Prior to NGS, RNA molecules are initially fragmented into short pieces of approximately 300 nt. In contrast, full-length RNA molecules are either sequenced directly or converted to cDNA before sequencing. Our further analyses demonstrate that the length of RNA-seq reads affects alignment accuracy, shorter reads are more susceptible to incorrect mapping to the reference genome, resulting in a lower estimation of A-to-I editing level when NGS RNA-seq employs shorter reads (Fig. 7). Since the quantification of A-to-I editing levels predominantly relies on NGS, both the levels and functions of A-to-I editing sites are likely to be lower estimated. Hence, to accurately measure A-to-I editing levels, it is essential to develop new high-throughput sequencing technologies or bioinformatic methods. In addition, the analyses of splicing or gene expression conducted using NGS might also be miscalculated due to alignment issues, which require further investigation.

It is widely believed that the per-base error rate of LRS is higher than NGS. Several analyses were conducted to assess whether the disadvantage of LRS affects the LRS/NGS difference on A-to-I RNA editing level quantification. Firstly, we raised the base quality cutoff of LRS as 10, consistently, NGS still showed lower estimates of A-to-I RNA editing levels compared to LRS (Figure S4). Secondly, while the effect of disadvantages of LRS is limited with high sequencing depth, we raised the coverage to 40, 50 and 60 and reanalyzed the comparison between NGS and LRS, and the consistent results were identified (Fig. 2A-B and Figure S3). For the full-length DNA/RNA amplicon sequencing, LRS was employed. Thirdly, for DNA amplicon sequencing, except one site, which may both a SNP and A-to-I editing site, most the A-to-I RNA editing levels are lower than 0.5% while half are 0% (Table S2), very close to the theoretical value of 0%, reflecting that the effect of LRS disadvantage on A-to-I RNA editing quantification is very limited. Additionally, the measurement of A-to-I RNA editing levels using LRS with a minimal base quality of 7 is consistent with the results via full-length RNA amplicon sequencing with minimal base quality 30 (Fig. 2C and Table S2). Collectively, the LRS/NGS difference should be real and does not attribute to the disadvantages of LRS, such as higher per-base error rates. However, we recommend that researchers verify measurements of A-to-I RNA editing sites intended for functional studies using one or more methods, such as full-length DNA or RNA amplicon sequencing.

## Supplementary Information

Supplementary Material 1.

Supplementary Material 2.

Supplementary Material 3.

Supplementary Material 4.

Supplementary Material 5.

Supplementary Material 6.

## Data Availability

Our raw RNA-seq dataset was uploaded to GEO with accession GSE318137. And the Python scripts and full-length amplicon sequencing data were uploaded to GitHub (https://github.com/Songlab2022/A-to-I-RNA-editing-quantification-comparison?tab=readme-ov-file#a-to-i). All the data now is available to referees.
