# Supplementary material for: Short-read RNA-seq yields lower estimates of A-to-I RNA editing levels than long-read cDNA sequencing
Source: Adv Biotechnol (Singap). 2026 Jul 22;4(3):27. doi: 10.1007/s44307-026-00123-w (PMC13391985; doi:10.1007/s44307-026-00123-w)
Supplement: Supplementary file 1 — Supplementary Material 1. [file 44307_2026_123_MOESM1_ESM.docx]

**
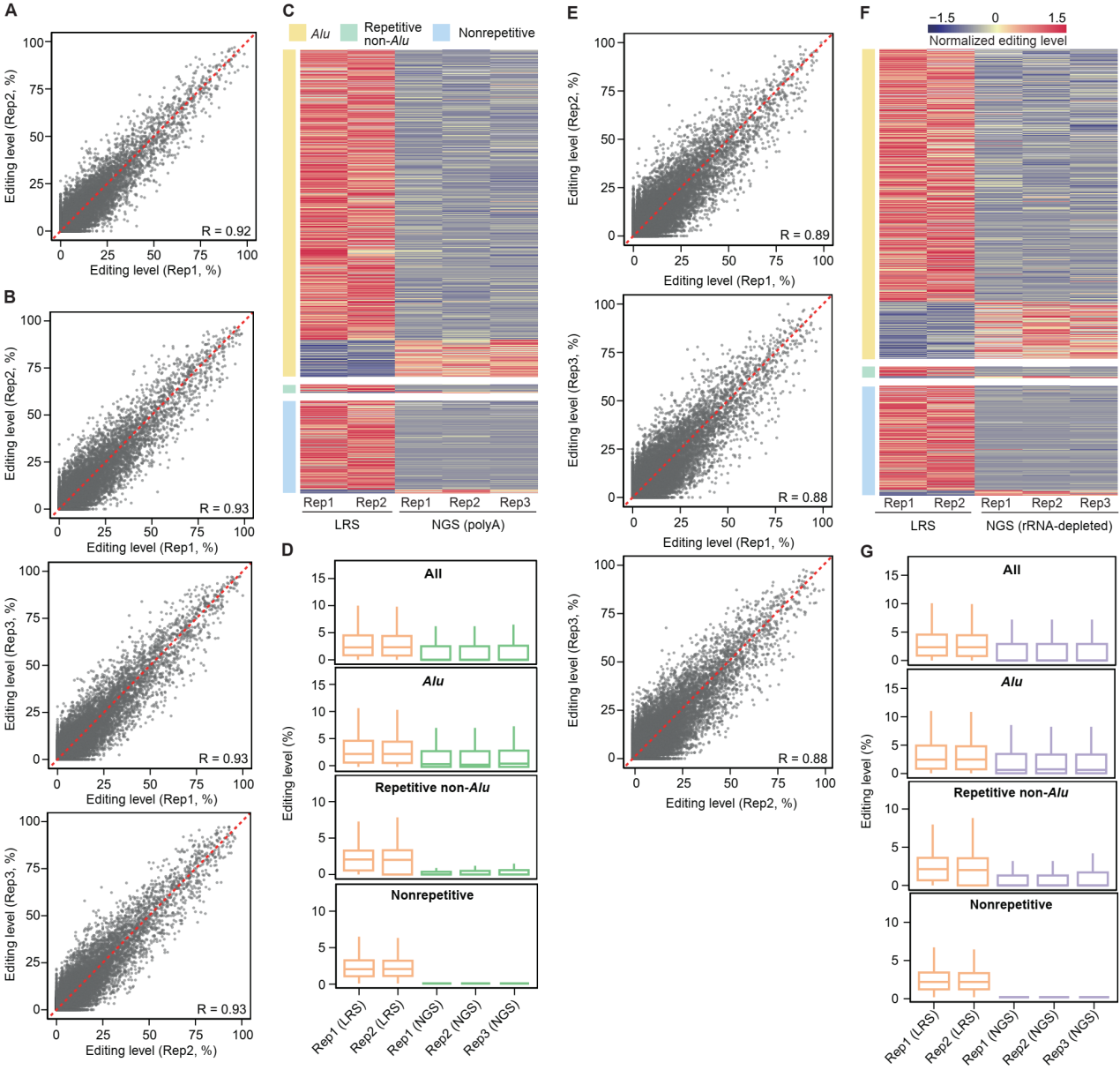
**

**Fig. S1** The quantification comparison of A-to-I editing levels between LRS, NGS polyA-selected and rRNA-depleted RNA-seq in HEK293T cells. **A** The correlation of A-to-I editing levels quantified via the two replicates of LRS polyA-selected cDNA RNA-seq in HEK293T cells. **B** The correlation of A-to-I editing levels quantified via the three replicates of NGS polyA-selected cDNA RNA-seq in HEK293T cells. **C-D** Heatmap (**C**) and boxplot (**D**) of the quantification comparison of A-to-I editing levels between via LRS polyA-selected and NGS polyA-selected cDNA RNA-seq in HEK293T cells. **E** The correlation of A-to-I editing levels quantified via the three replicates of NGS rRNA-depleted cDNA RNA-seq in HEK293T cells. **F-G** Heatmap (**F**) and boxplot (**G**) of the quantification comparison of A-to-I editing levels between via LRS polyA-selected and NGS rRNA-depleted cDNA RNA-seq in HEK293T cells.

**
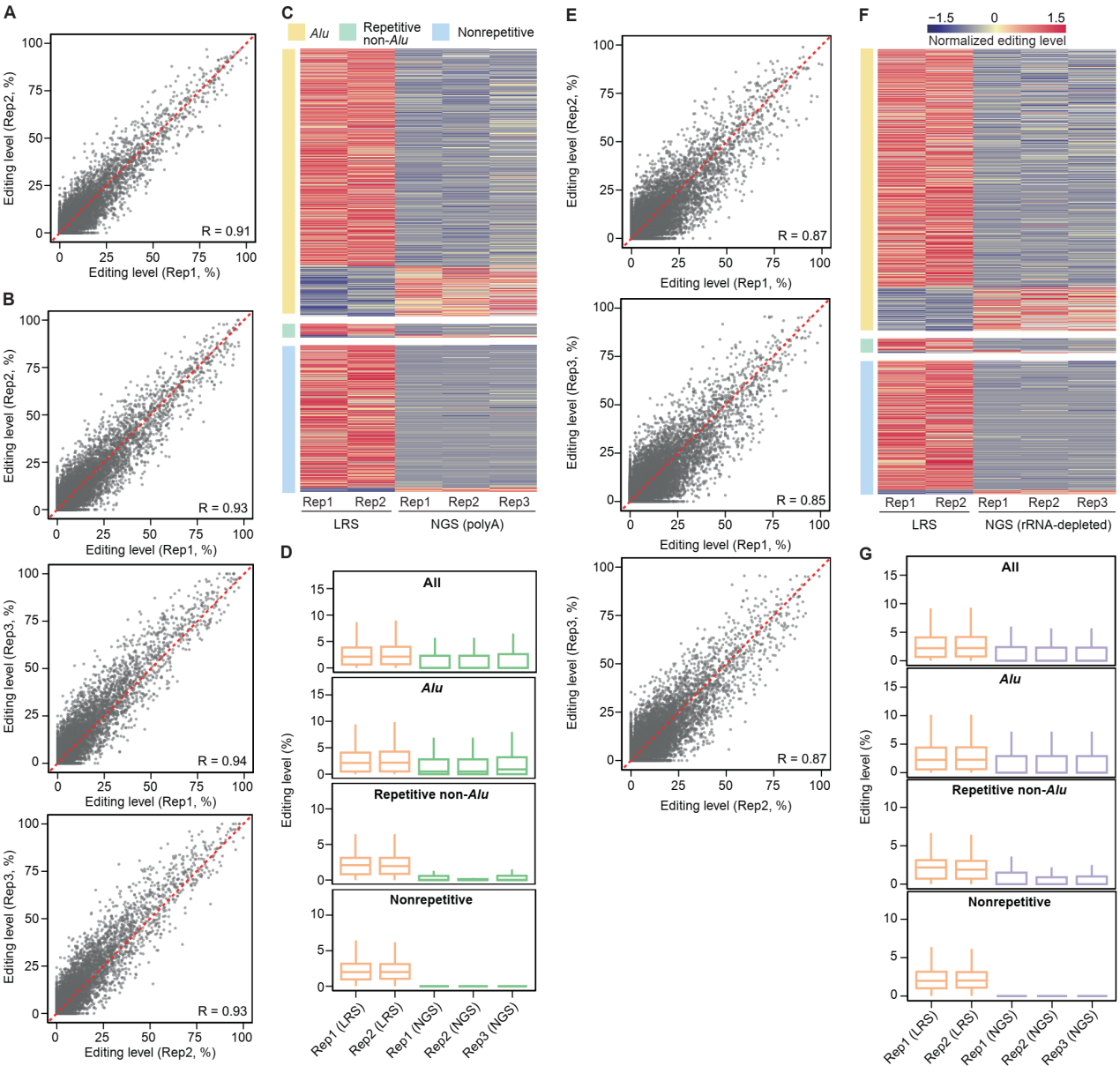
**

**Fig. S2** The quantification comparison of A-to-I editing levels between LRS, NGS polyA-selected and rRNA-depleted RNA-seq in U2OS cells. **A** The correlation of A-to-I editing levels quantified via the two replicates of LRS polyA-selected cDNA RNA-seq in U2OS cells. **B** The correlation of A-to-I editing levels quantified via the three replicates of NGS polyA-selected cDNA RNA-seq in U2OS cells. **C-D** Heatmap (**C**) and boxplot (**D**) of the quantification comparison of A-to-I editing levels between via LRS polyA-selected and NGS polyA-selected cDNA RNA-seq in U2OS cells. **E** The correlation of A-to-I editing levels quantified via the three replicates of NGS rRNA-depleted cDNA RNA-seq in U2OS cells. **F-G** Heatmap (**F**) and boxplot (**G**) of the quantification comparison of A-to-I editing levels between via LRS polyA-selected and NGS rRNA-depleted cDNA RNA-seq in U2OS cells.

**
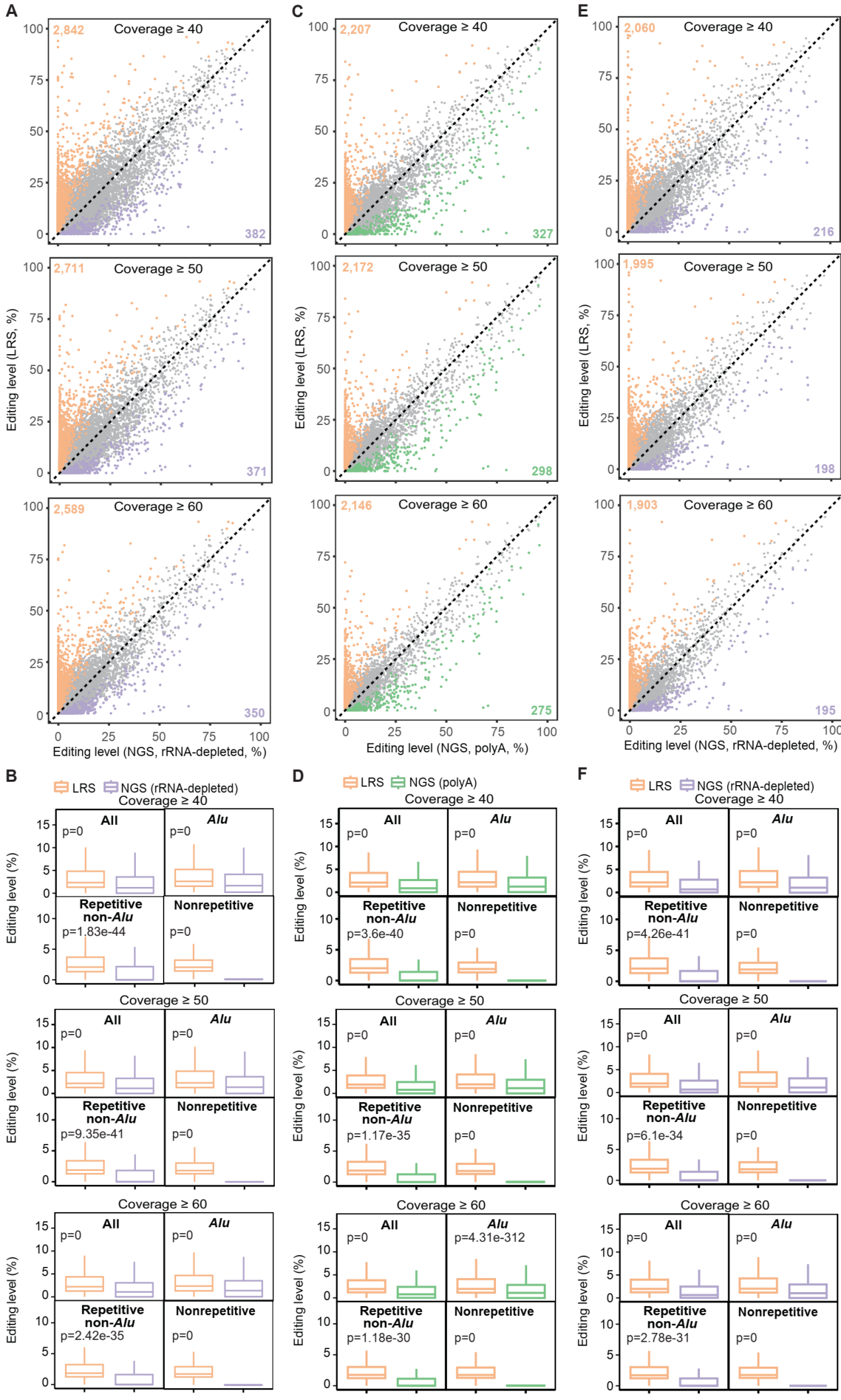
**

**Fig. S3** Quantification comparison of A-to-I editing levels between via LRS and NGS cDNA RNA-seq with different coverage filter. **A-B** Dotplot (**A**) and boxplot (**B**) of the quantification comparison of A-to-I editing levels between via LRS and NGS rRNA-depleted cDNA RNA-seq in HEK293T cells with different coverage filter. **C-D** Dotplot (**C**) and boxplot (**D**) of the quantification comparison of A-to-I editing levels between via LRS and NGS polyA-selected cDNA RNA-seq in U2OS cells with different coverage filter. **E-F** Dotplot (**E**) and boxplot (**F**) of the quantification comparison of A-to-I editing levels between via LRS and NGS rRNA-depleted cDNA RNA-seq in U2OS cells with different coverage filter. Only the A-to-I editing site with FDR < 0.05 was defined as significant, and p values were calculated using the Mann-Whitney U test.

**
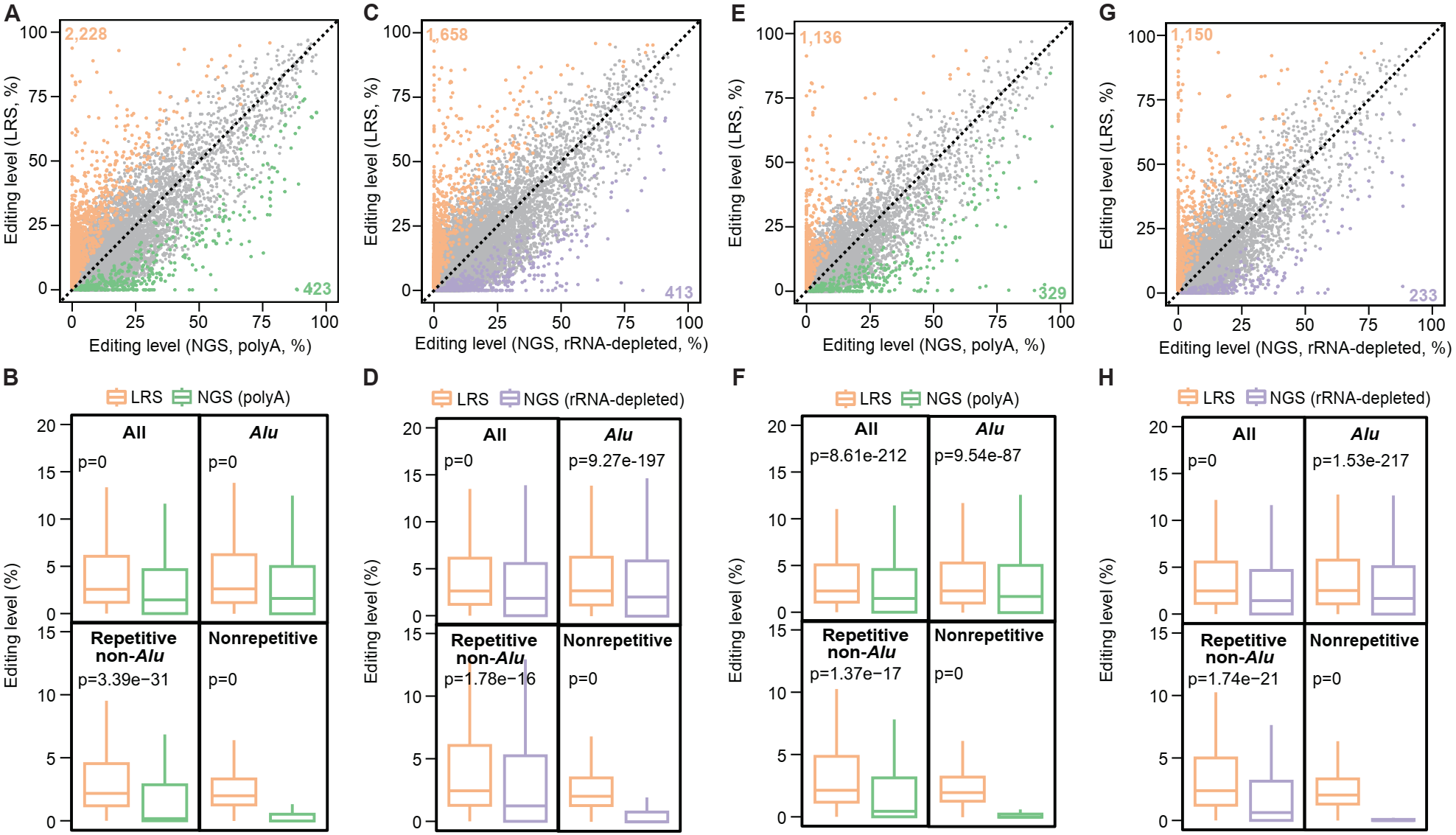
**

**Fig. S4** Quantification comparison of A-to-I editing levels between via NGS and LRS cDNA RNA-seq with higher base quality cutoff (base quality of LRS ≥ 10). **A-B** Dotplot (**A**) and boxplot (**B**) of the quantification comparison of A-to-I editing levels between via LRS and NGS polyA-selected cDNA RNA-seq in HEK293T cells. **C-D** Dotplot (**C**) and boxplot (**D**) of the quantification comparison of A-to-I editing levels between via LRS and NGS rRNA-depleted cDNA RNA-seq in HEK293T cells. **E-F** Dotplot (**E**) and boxplot (**F**) of the quantification comparison of A-to-I editing levels between via LRS and NGS polyA-selected cDNA RNA-seq in U2OS cells. **G-H** Dotplot (**G**) and boxplot (**H**) of the quantification comparison of A-to-I editing levels between via LRS and NGS rRNA-depleted cDNA RNA-seq in U2OS cells. Only the A-to-I editing site with FDR < 0.05 was defined as significant, and p values were calculated using the Mann-Whitney U test.

**
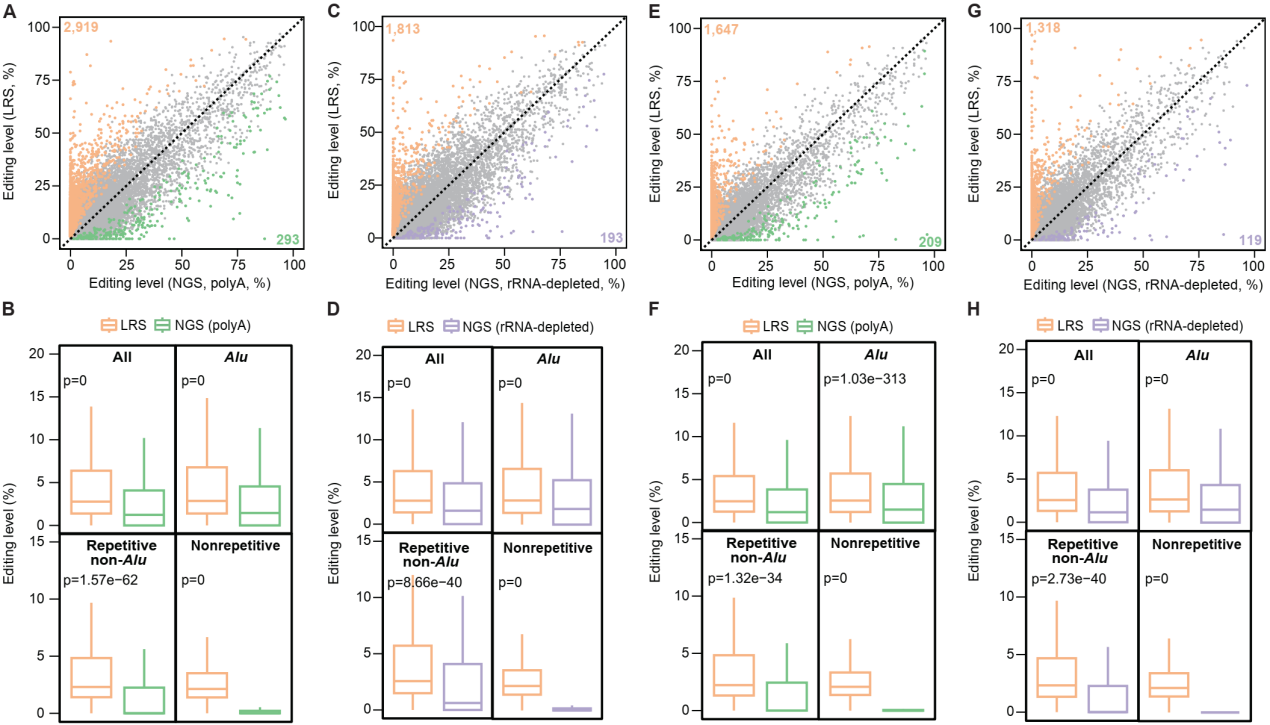
**

**Fig. S5** Quantification comparison of A-to-I editing levels between via NGS and LRS cDNA RNA-seq without PCR duplicate reads. **A-B** Dotplot (**A**) and boxplot (**B**) of the quantification comparison of A-to-I editing levels between via LRS and NGS polyA-selected cDNA RNA-seq in HEK293T cells. **C-D** Dotplot (**C**) and boxplot (**D**) of the quantification comparison of A-to-I editing levels between via LRS and NGS rRNA-depleted cDNA RNA-seq in HEK293T cells. **E-F** Dotplot (**E**) and boxplot (**F**) of the quantification comparison of A-to-I editing levels between via LRS and NGS polyA-selected cDNA RNA-seq in U2OS cells. **G-H** Dotplot (**G**) and boxplot (**H**) of the quantification comparison of A-to-I editing levels between via LRS and NGS rRNA-depleted cDNA RNA-seq in U2OS cells. Only the A-to-I editing site with FDR < 0.05 was defined as significant, and p values were calculated using the Mann-Whitney U test.

**
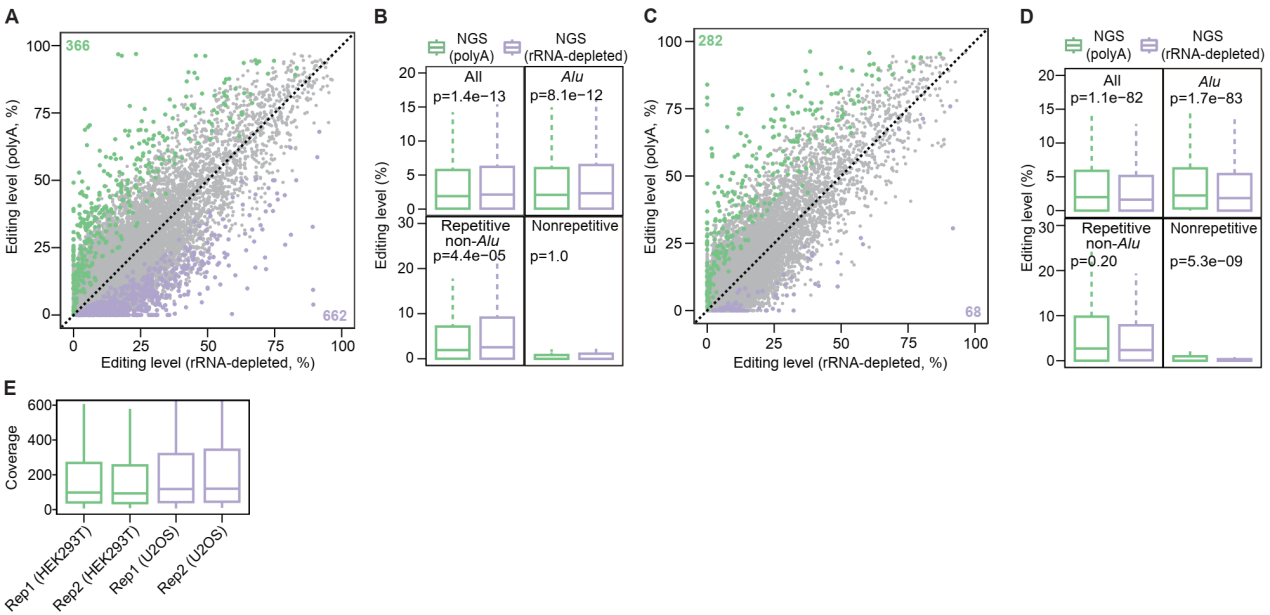
**

**Fig. S6** The quantification comparison of A-to-I editing levels between NGS polyA-selected and rRNA-depleted RNA-seq in HEK293T or U2OS cells. **A-B** Dotplot (**A**) and boxplot (**B**) of the quantification comparison of A-to-I editing levels between NGS polyA-selected and rRNA-depleted cDNA RNA-seq in HEK293T cells. **C-D** Dotplot (**C**) and boxplot (**D**) of the quantification comparison of A-to-I editing levels between NGS polyA-selected and rRNA-depleted cDNA RNA-seq in U2OS cells. **E** The coverage distribution of LRS used for quantifying A-to-I RNA editing levels in this study. Only the A-to-I editing site with FDR < 0.05 was defined as significant, and p values were calculated using the Mann-Whitney U test.

**
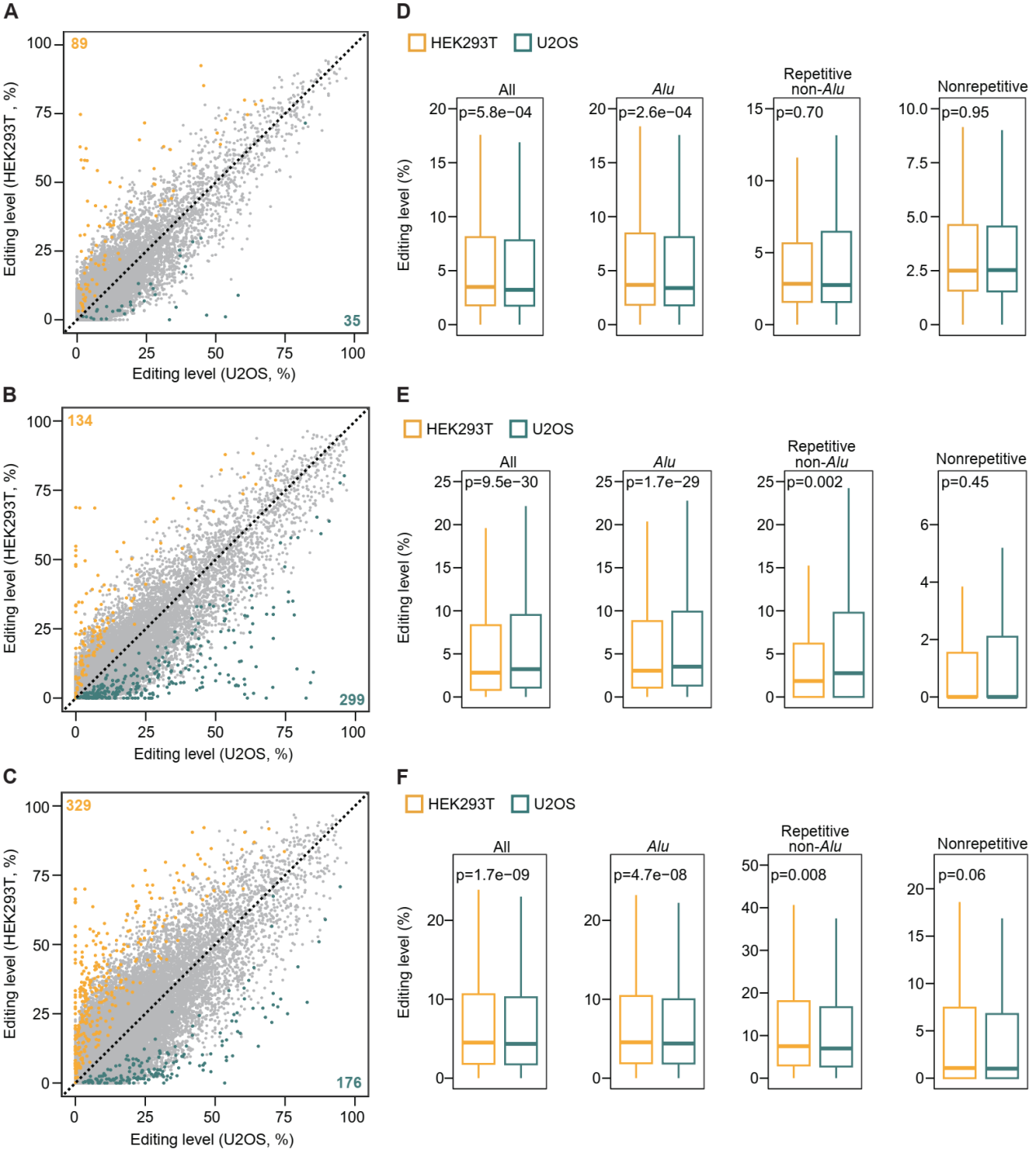
**

**Fig. S7** The quantification comparison of A-to-I editing levels between HEK293T and U2OS cell lines using LRS polyA-selected, NGS polyA-selected or rRNA-depleted cDNA RNA-seq. **A-C** Dotplot of the quantification comparison of A-to-I editing levels between HEK293T and U2OS cells using LRS polyA-selected (**A**), NGS polyA-selected (**B**) or rRNA-depleted (**C**) cDNA RNA-seq, only the A-to-I editing site with FDR < 0.05 was defined as significant. **D-F** Boxplot of the quantification comparison of A-to-I RNA editing levels between HEK293T and U2OS cells using LRS polyA-selected (**D**), NGS polyA-selected (**E**) or rRNA-depleted (**F**) cDNA RNA-seq, and p values were calculated using the Mann-Whitney U test.

**
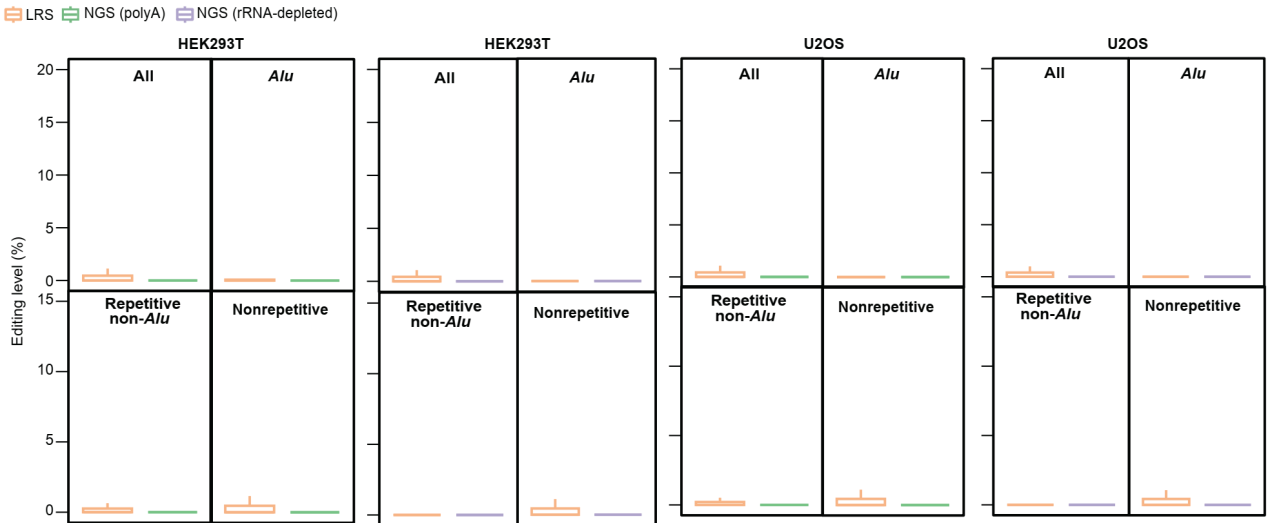
**

**Fig. S8** The level quantification comparison of unedited A candidates between LRS and NGS.

**
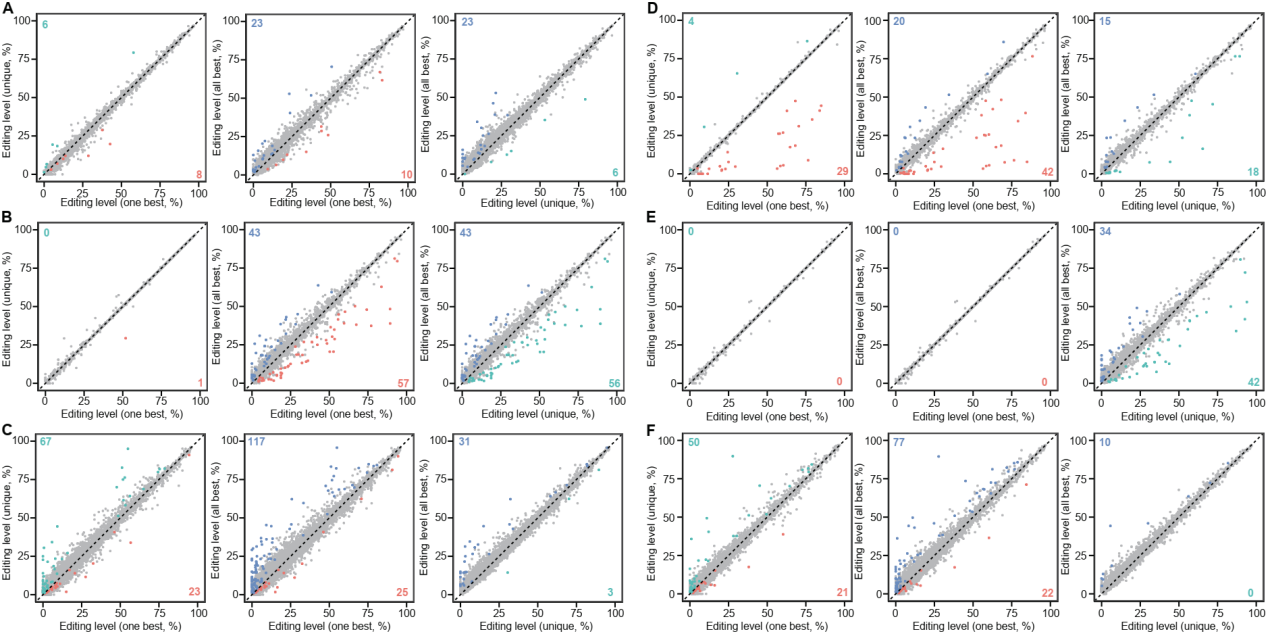
**

**Fig. S9** The quantification comparison of A-to-I editing levels between different alignment strategies using LRS or NGS RNA-seq performed in this study. **A-C** The quantification comparison of A-to-I editing levels between any two of these three alignment strategies of HEK293T cells using NGS polyA-selected (**A**), rRNA-depleted (**B**) or LRS (**C**) cDNA RNA-seq. **D-F** The quantification comparison of A-to-I editing levels between any two of these three alignment strategies of U2OS cells using NGS polyA-selected (**D**), rRNA-depleted (**E**) or LRS (**F**) cDNA RNA-seq. The three different alignment strategies: unique: utilizing only uniquely mapped hits; one best: combining uniquely mapped hits with one of the multiply mapped hits that has the best mapping scores; all best: using both the uniquely mapped hits and all the multiply mapped hits with the best mapping scores. P values were calculated using the Chi-Square Test, and only the A-to-I editing site with p value < 0.05 was defined as significant.

**
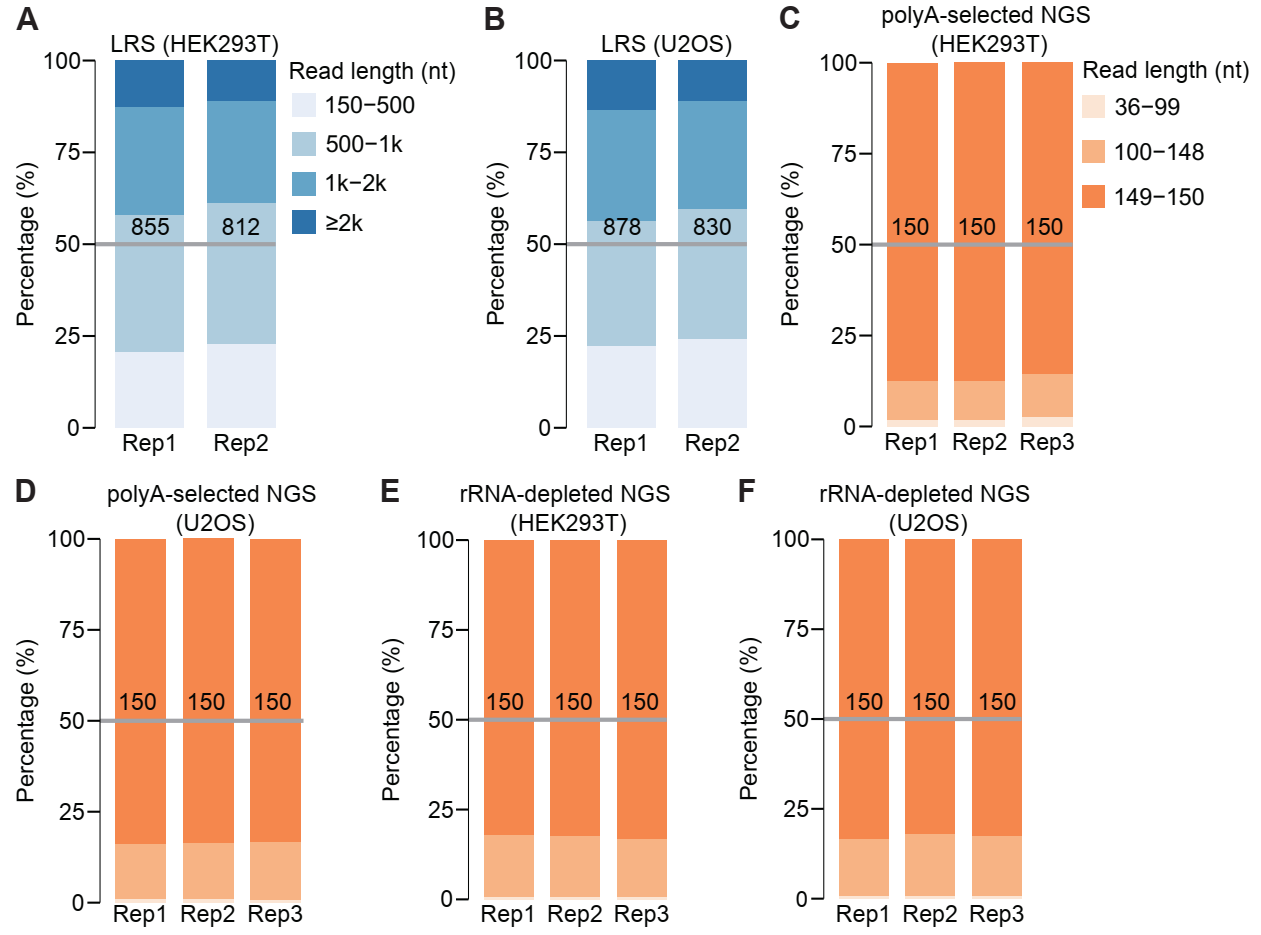
**

**Fig. S10** The distribution of filtered read length of LRS and NGS RNA-seq performed in this study. **A-B** The distribution of read lengths of LRS RNA-seq in HEK293T (**A**) and U2OS (**B**) cells. **C-D** The distribution of read lengths of NGS polyA-selected RNA-seq in HEK293T (**C**) and U2OS (**D**) cells. **E-F** The distribution of read lengths of NGS rRNA-depleted RNA-seq in HEK293T (**E**) and U2OS (**F**) cells.

**
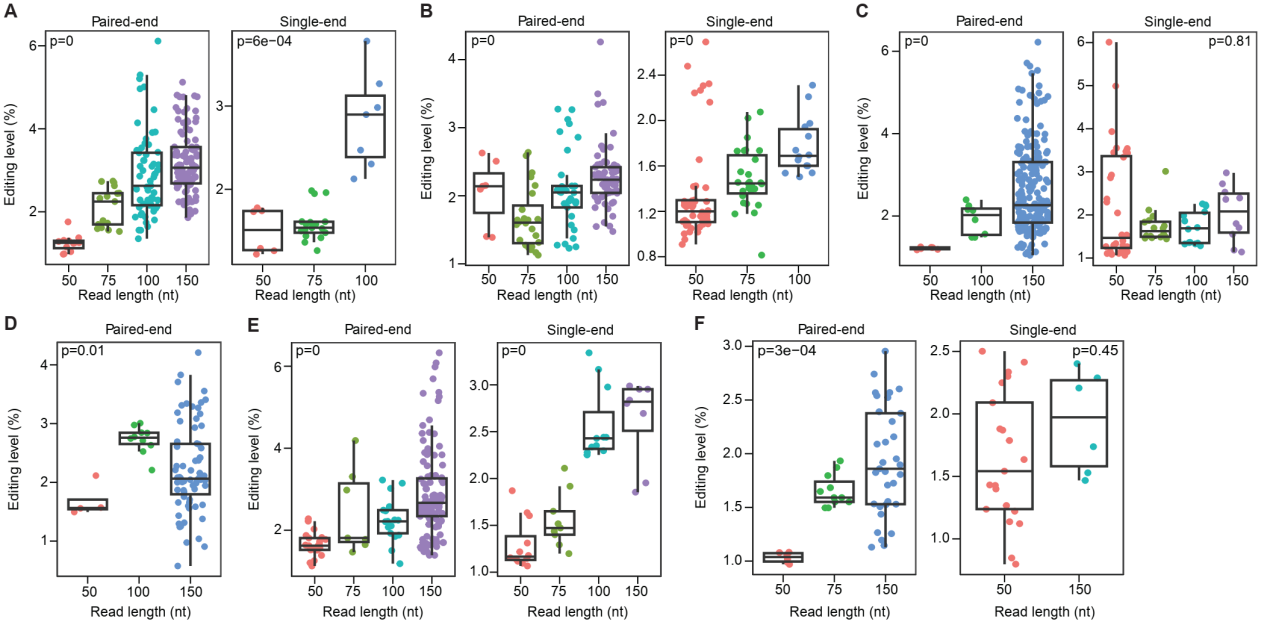
**

**Fig. S11** The quantification comparison of A-to-I editing levels among different read length of NGS cDNA RNA-seq in custom cell lines. **A** U2OS; **B** HeLa; **C** HepG2; **D** K562; **E** A549; **F** U87. P values were calculated using the Kruskal-Wallis H test.

**
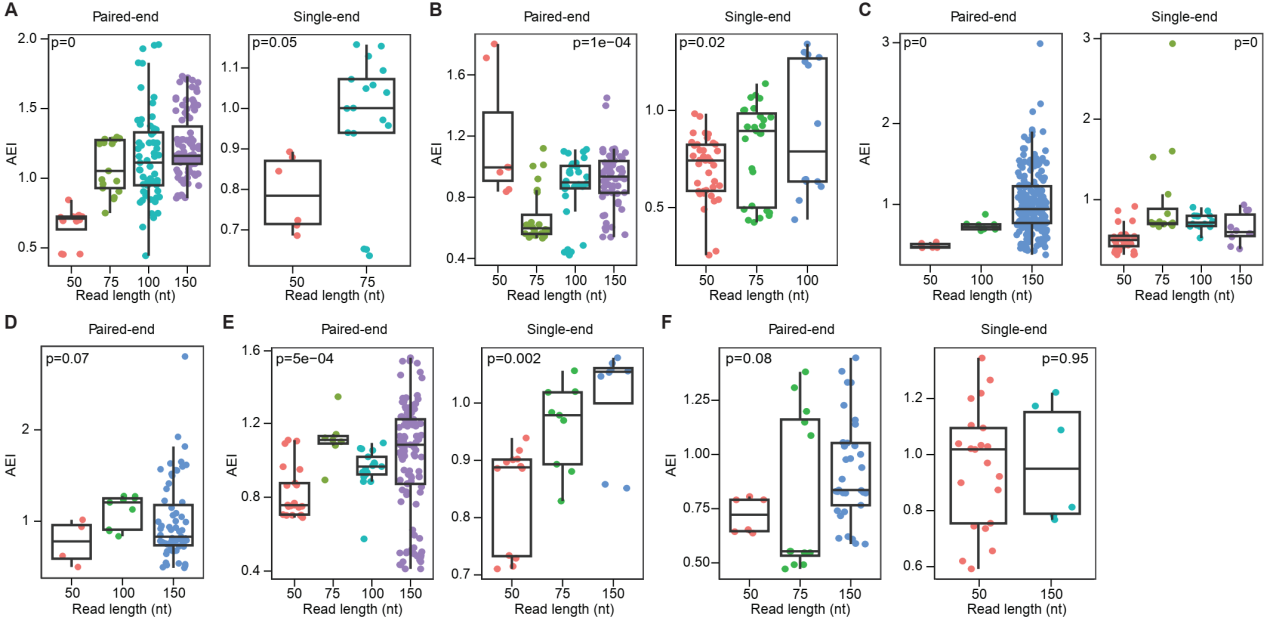
**

**Fig. S12** The quantification comparison of AEI among different read lengths of NGS cDNA RNA-seq in custom cell lines. **A** U2OS; **B** HeLa; **C** HepG2; **D** K562; **E** A549; **F** U87. P values were calculated using the Kruskal-Wallis H test.


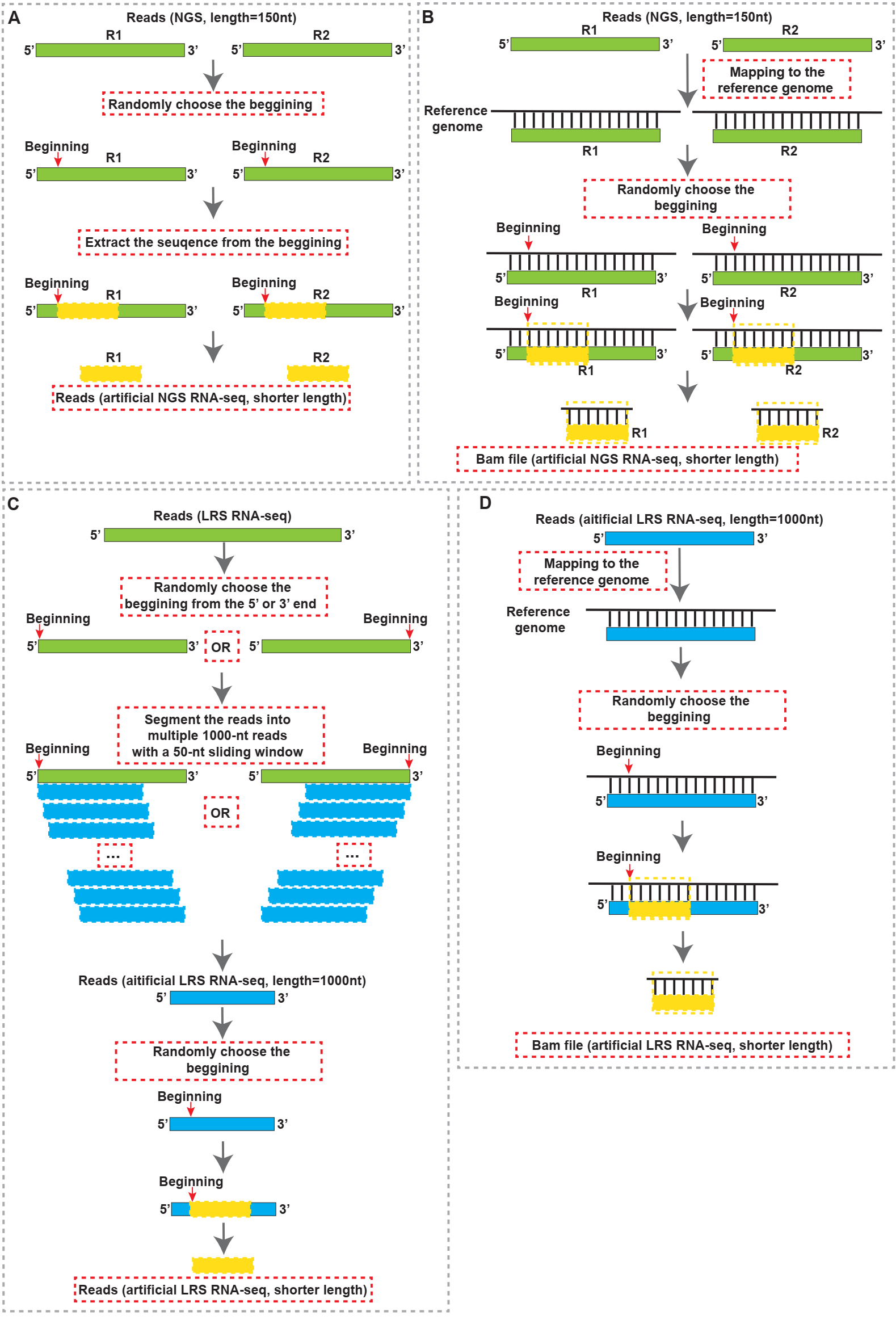


**Fig. S13** The schematic diagram of constructing artificial NGS or LRS cDNA RNA-seq with different read lengths. **A-B** The schematic diagram of constructing artificial NGS cDNA RNA-seq for fastq (**A**) or bam (**B**); **C-D** The schematic diagram of constructing artificial LRS cDNA RNA-seq for fastq (**C**) or bam (**D**).

**
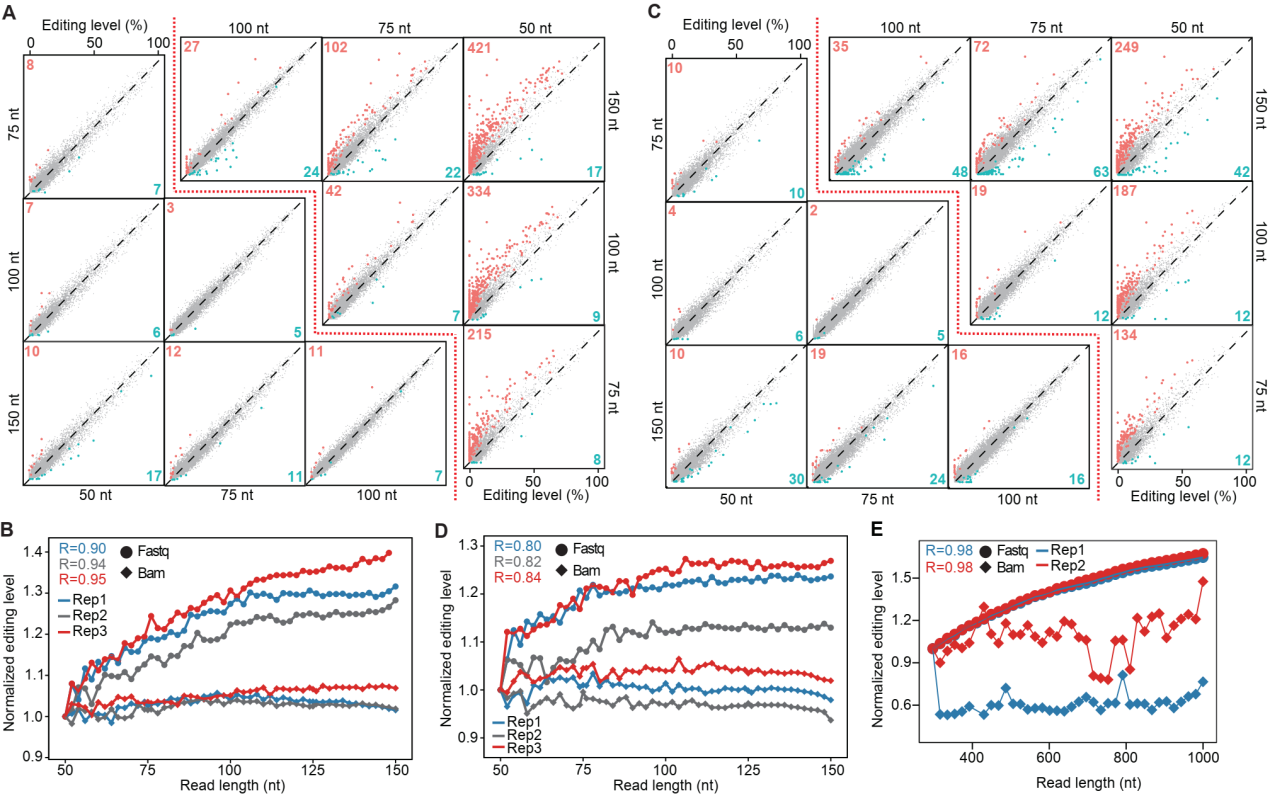
**

**Fig. S14** The quantification comparison of A-to-I editing levels among different read lengths of artificial NGS or LRS RNA-seq in U2OS cell line. **A** Dotplot of the quantification comparison of A-to-I editing levels between artificial NGS polyA-selected cDNA RNA-seq with different read lengths in U2OS cells. The upper-right represents the artificial RNA-seq derived from the fastq, while the bottom-left represents the artificial RNA-seq derived from the bam. **B** The correlation between the normalized quantification of A-to-I editing levels and the read lengths of artificial NGS polyA-selected cDNA RNA-seq in U2OS cells. The editing level of each sample was normalized by dividing editing level of the RNA-seq with 50-nt read length. **C** Dotplot of the quantification comparison of A-to-I editing levels between artificial NGS rRNA-depeted cDNA RNA-seq with different read lengths in U2OS cells. The upper-right represents the artificial RNA-seq derived from the fastq, while the bottom-left represents the artificial RNA-seq derived from the bam. The three replicates were merged. **D** The correlation between the normalized quantification of A-to-I editing levels and the read lengths of artificial NGS rRNA-depleted cDNA RNA-seq in U2OS cells. The editing level of each sample was normalized by dividing editing level of the RNA-seq with 50-nt read length. **E** The correlation between the normalized quantification of A-to-I editing levels and the read lengths of artificial LRS polyA-selected cDNA RNA-seq in U2OS cells. The editing level of each sample was normalized by dividing editing level of the RNA-seq with 50-nt read length. P values were calculated using the Mann-Whitney U test and p value < 0.05 was defined as significant.

**
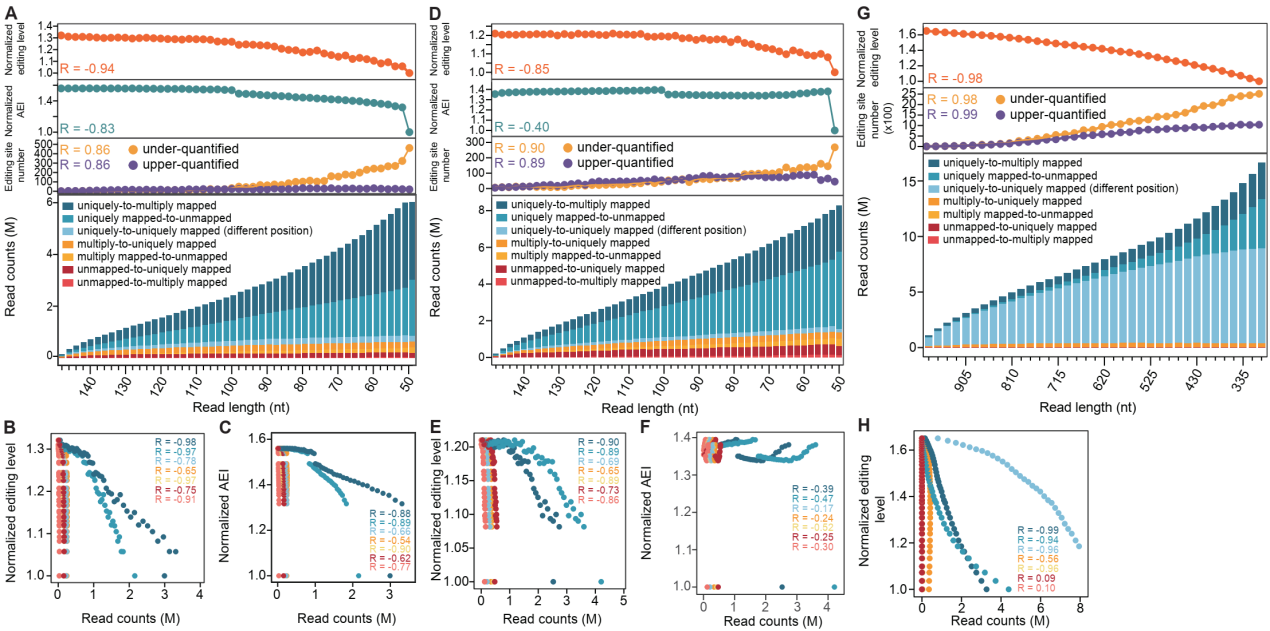
**

**Fig. S15** The correlation between A-to-I editing level quantification and inaccurate alignment types among different read lengths of artificial NGS or LRS RNA-seq in U2OS cell line. **A** The normalized editing level, AEI, significantly under- or upper-quantified editing site number and inaccurately aligned read count among the read lengths of artificial NGS polyA-selected cDNA RNA-seq of U2OS cells. **B** The correlation between normalized editing level and inaccurately aligned read count of artificial NGS polyA-selected cDNA RNA-seq of U2OS cells. **C** The correlation between normalized AEI and inaccurately aligned read count of artificial NGS polyA-selected cDNA RNA-seq of U2OS cells. **D** The normalized editing level, AEI, significantly under- or upper-quantified editing site number and inaccurately aligned read count among the read lengths of artificial NGS rRNA-depleted cDNA RNA-seq of U2OS cells. **E** The correlation between normalized editing level and inaccurately aligned read count of artificial NGS rRNA-depleted cDNA RNA-seq of U2OS cells. **F** The correlation between normalized AEI and inaccurately aligned read count of artificial NGS rRNA-depleted cDNA RNA-seq of U2OS cells. **G** The normalized editing level, significantly under- or upper-quantified editing site number and inaccurately aligned read count among the read lengths of artificial LRS polyA-selected cDNA RNA-seq of U2OS cells. **H** The correlation between normalized editing level and inaccurately aligned read count of artificial LRS polyA-selected cDNA RNA-seq of U2OS cells.

**
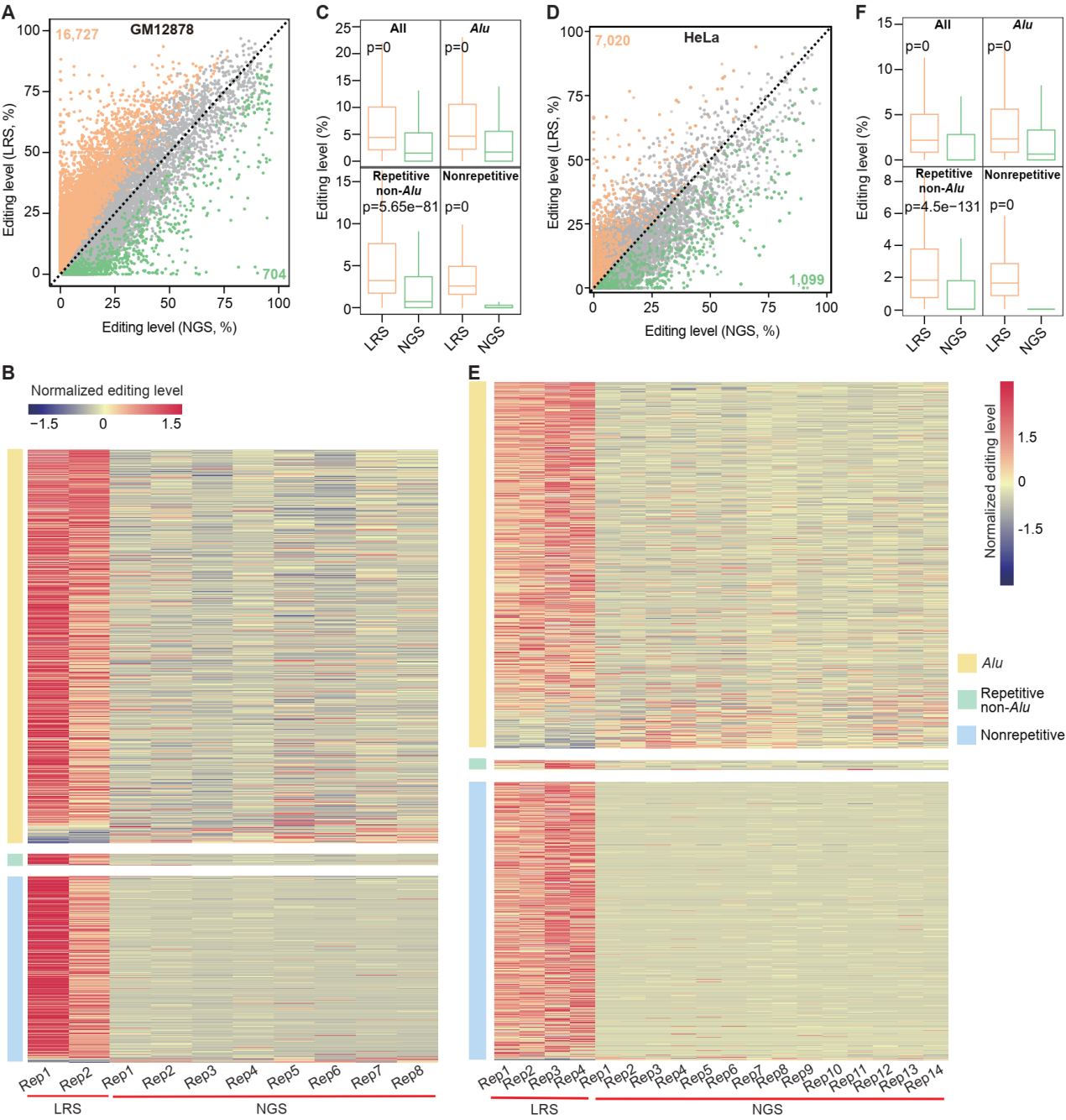
**

**Fig. S16** The quantification comparison of A-to-I editing levels between cross-study LRS and NGS cDNA RNA-seq in human cancer cell lines. **A-C** Dotplot (**A**), heatmap (**B**) and boxplot (**C**) of the quantification comparison of A-to-I editing levels between LRS and NGS cDNA RNA-seq of GM12878 cells. **D-F** Dotplot (**D**), heatmap (**E**) and boxplot (**F**) of the quantification comparison of A-to-I editing levels between LRS and NGS cDNA RNA-seq of HeLa cells. Only the A-to-I editing site with FDR < 0.05 was defined as significant, and p values were calculated using the Mann-Whitney U test.


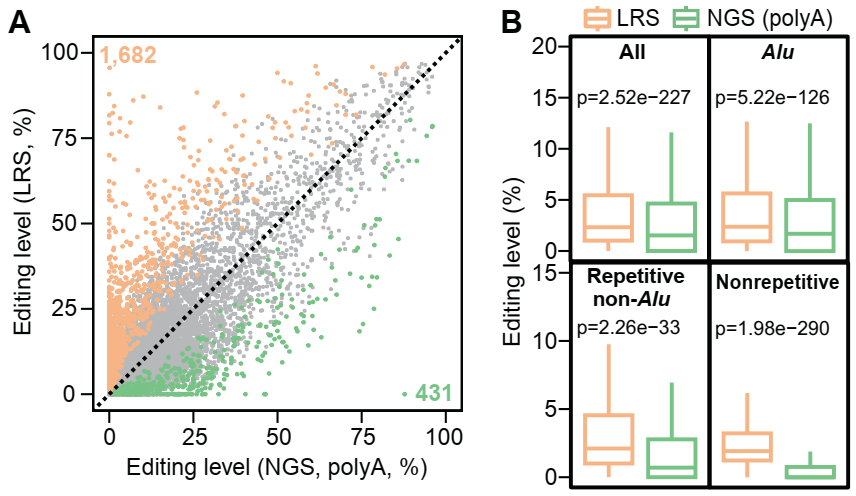


**Fig. S17** The quantification comparison of A-to-I editing levels between LRS and NGS polyA-selected cDNA RNA-seq using GRCh38 in HEK293T. Dotplot (**A**) and boxplot (**B)** of LRS/NGS comparison. FDR < 0.05 was used to define significantly different sites. P values were calculated using the Mann-Whitney U test.

**Table S1** The statistics of NGS and LRS RNA-seq performed in this study.

**Table S2** The comparison of A-to-I RNA editing quantification among LRS, NGS, amplicon (RNA) and amplicon (DNA).

**Table S3** The list of NGS RNA-seq used in Fig. 3, Figures S11 and S12.

**Table S4** The list of NGS and LRS RNA-seq data used in Fig. 6.

**Table S5** The list of NGS and LRS RNA-seq data used in Figure S16.
